# Chemoenzymatic Route toward a De Novo Enantioselective Total Synthesis of (*S*)‐Baclofen Based on Metal‐Catalyzed Hydroformylation and Enzymatic Transamination

**DOI:** 10.1002/cbic.202500108

**Published:** 2025-07-04

**Authors:** Feodor Belov, Hannah Bork, Luise Hänel, Manideep V. Kollipara, Matthias Höhne, Harald Gröger, Jan von Langermann

**Affiliations:** ^1^ Institute of Chemistry Biocatalytic Synthesis Group Otto von Guericke University of Magdeburg Building 28, Universitätsplatz 2 39106 Magdeburg Germany; ^2^ Faculty of Chemistry Bielefeld University Universitätsstrasse 25 33615 Bielefeld Germany; ^3^ Institute of Biochemistry Protein Biochemistry Group University of Greifswald Felix‐Hausdorff‐Str. 4 17489 Greifswald Germany; ^4^ Institute of Chemistry Biocatalysis Group Technical University of Berlin Müller‐Breslau‐Strasse 10 10623 Berlin Germany

**Keywords:** amines, biocatalysis, cascade, chiral, homogeneous catalysis

## Abstract

This study explores the chemoenzymatic synthesis of (*S*)‐baclofen, which involves a sequential combination of transition metal catalysis and biocatalysis. The synthesis approach starts from a readily accessible cinnamic acid ester that is converted using a rhodium‐based hydroformylation catalyst toward the corresponding chiral aldehyde. This compound is subsequently converted via a transaminase‐catalyzed reaction system that yields the desired β‐chiral amino acid ester and the final free β‐chiral amino acid (*s* )‐baclofen after a simple hydrolysis reaction. This synthesis concept does provide high atom efficiency and does not require an additional chiral resolution step of a racemic product.

## Graphical abstract

A unique chemoenzymatic synthesis route toward (*S*)‐baclofen is presented, which highlights a hydroformylation and biocatalysis step toward the synthesis of such a challenging β‐chiral amino acid. The shown methodology exhibits high conversions and enantiomeric excess and is transferable to other β‐chiral amines and amino acids.

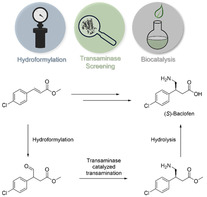

## 1 Introduction

The task of producing pharmaceuticals comprises a multitude of challenges for modern synthetic organic chemistry, which starts with the design of an “ideal” retrosynthetic strategy being capable, besides other benefits, to minimize reaction steps when starting from cheap and readily available raw materials.^[^ ^1^ ^]^ Other criteria to be matched are excellent product quality, low production costs, sustainability, and scalability.^[^ ^2^ ^]^ Furthermore, rather than being a “single product solution,” such a developed process ideally shows the feasibility to be extended toward a technology platform being suitable to produce a broad range of related compounds on scale.^[^ ^3^ ^]^ Particular chances for achieving these goals arise from utilizing the full spectrum of catalytic methods, including chemocatalysis and biocatalysis, as well as process engineering techniques.^[^ ^4^ ^]^ Although this conclusion and such process goals seem logical and even trivial, they have often not yet become realized in practice.^[^ ^5^ ^]^ In particular, the integration of biocatalysis as a tool in organic synthesis labs often still faces a substantial entrance barrier for chemists. Obviously, overcoming such hurdles would enable unique novel retrosynthetic pathways toward challenging pharmaceutical compounds with enormous synthetic benefits in terms of shortened synthesis routes, reducing the amount of waste and increasing overall yield, thus contributing to improved economy as well as sustainability of the overall process.^[^ ^6^, ^7^ ^]^

In particular, for chiral amine synthesis, transaminases have proven to be a valuable tool with multiple established applications, including processes in the pharmaceutical industry.^[^ ^8^ ^]^

**Scheme 1:**
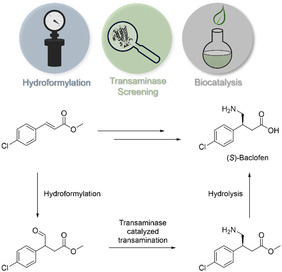
Chemoenzymatic synthesis concept toward (*S*)‐baclofen involving hydroformylation and a transaminase‐catalyzed reaction.

In the following study we showcase how exactly such a combination of complementary technologies from these different worlds of chemocatalysis and biocatalysis can lead to an efficient synthetic strategy with potential for industrial application, exemplified for the general class of β‐chiral amino acids. In particular, we demonstrate this for one enantiomer of baclofen (**Scheme** **1**), which is an important drug used for muscle spasticity treatment and other medical applications.^[^ ^9^ ^]^ Our chemoenzymatic strategy starts with the hydroformylation of a readily available cinnamic acid ester, resulting in the corresponding chiral aldehyde. The subsequent transaminase‐catalyzed reaction then yields the intermediate (*S*)‐baclofen ester, which, in part, undergoes partial cyclization to a lactam and eventually hydrolysis as the free β‐chiral amino acid and final product (*S*)‐baclofen.

## 2 Results and Discussion

### 2.1 Hydroformylation of Cinnamic Acid Derivatives toward Baclofen Precursors

**Scheme 2:**
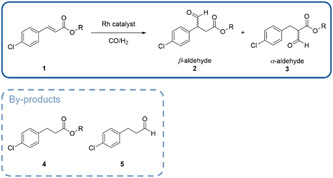
Reaction scheme for the hydroformylation of 4‐chlorocinnamates (**1a**) with possible by‐products. An additional by‐product **6** was observed with <3% (see Supporting Information).

The first step in the synthetic strategy for the production of baclofen is a rhodium‐catalyzed hydroformylation starting from the corresponding α,β‐unsaturated 4‐chlorocinnamate (**1**). In general, hydroformylation is a widely applied technology with various large‐scale applications for conversion of alkenes with syngas toward aldehydes,^[^ ^10^, ^11^ ^]^ and in our previous work we reported the spectroscopic investigation of the hydroformylation of methyl 4‐chlorocinnamate with a commercial sample and showed a process development including the optimization using a design of experiments approach. 4‐Chlorocinnamate esters (**1**) can be viewed as derivatives of either styrene or acrylates and represent a challenging substrate regarding the desired regioselectivity in the hydroformylation. With different unmodified Rh complexes, the β‐regioselective hydroformylation was observed.^[^ ^12^, ^13^ ^]^ This preferred selectivity served as the basis for this work, which focuses on the applicability and scale‐up. As the catalyst, we employed a commercial sample of a Rh catalyst named “[HRh(PPh_3_)_4_],” which behaved like an unmodified Rh catalyst and showed comparable results to [Rh(acac)(CO)_2_].^[^ ^12^ ^]^ **Scheme** **2** shows the reaction pathway to the desired β‐aldehyde **2** and the regioisomeric α‐aldehyde **3.** The latter was also detected as the tautomeric enol, which is in accordance with the literature.^[^ ^14^ ^]^ Potentially formed by‐products are the hydrogenated 3‐(4‐chlorophenyl)propanoate (**4**) and 3‐(4‐chlorophenyl)propanal (**5**). The first is the most prevalent by‐product. In the following, the hydroformylations are evaluated according to the conversion, regioselectivity and selectivity to the β‐aldehyde **2.** The regioselectivity is defined as formation of the β‐aldehyde **2** over the sum of β‐ and α‐aldehydes **2** and **3**. The term selectivity is defined as the preferred β‐aldehyde **2** over all by‐products.

### 2.2 Substrate Screening and Substrate Loading

In terms of atom economy concerning the overall synthesis of baclofen, 4‐chlorocinnamic acid would be an ideal substrate for hydroformylation. However, its solubility in organic solvents is very limited. To determine the most suitable cinnamate, the ester residue of the hydroformylation substrate was modified from methyl to ethyl and isopropyl. Methyl 4‐chlorocinnamate (**1a**) and the ethyl and isopropyl esters **1b** and **1c** were synthesized from the corresponding acid through esterification. The hydroformylation under standard conditions (80 bar CO/H_2_ (1:1), 80 °C) resulted in a comparable conversion and regioselectivity for the methyl **1a**, ethyl **1b** and isopropyl ester **1c** (**Table** **1**).

**Table 1:** Hydroformylation of methyl 4‐chlorocinnamate (1a), ethyl 4‐chlorocinnamate (1b), and isopropyl 4‐chlorocinnamate (1c).

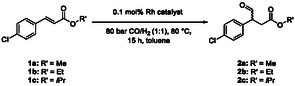					
**Entry**	Substrate	*R*'	Conversion [%]	Regioselectivity [%]a)	Selectivity [%]b)
**1**	**1a**	Me	78 ± 6	89 ± 2	68 ± 1
**2**	**1b**	Et	79 ± 12	91 ± 1	71 ± 1
**3**	**1c**	^*i*^Pr	91 ± 1	91 ± 1	75 ± 1

a) Reaction conditions: 0.2 m substrate, “[HRh(PPh_3_)_4_]” (obtained from TCI, according to ICP‐OES: 0.1 mol% Rh), toluene, 80 bar CO/H_2_ (1:1), 80 °C, 15 h, *V*_total_ = 5 mL, reactions performed in duplicate. Regioselectivity: Amount of β‐aldehyde **2** in relation to the sum of the amounts of β‐aldehyde **2** and α‐aldehyde **3** (in %); determined by ^1^H‐NMR spectroscopy; analogous for ethyl and isopropyl derivatives.

b) Selectivity: Amount of β‐aldehyde **2** in relation to the amounts of all detected formed products (**2**, **3**, **4**, **5**, **6**) (in %). By‐product **6a** was observed with ≤2%, while **6b** and **6c** were not detected.

**Scheme 3:**
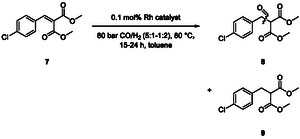
Hydroformylation of dimethyl 2‐(4‐chlorobenzy‐lidene)malonate (**7**). Reaction conditions: 0.20 m substrate, “[HRh(PPh_3_)_4_]” (TCI, according to ICP‐OES: 0.1 mol% Rh), toluene, 80 bar CO/H_2_ (5:1–1:2), 80 °C, 15–24 h, *V*_total_ = 3–5 mL, single point determination.

The hydroformylation of isopropyl 4‐chlorocinnamate (**1c**) showed the highest selectivity to the β‐aldehyde **2** of 75%, which was only slightly higher than that with methyl and ethyl groups. Thus, the residue seems to have a negligible influence on hydroformylation in terms of electronic or steric factors. For the overall baclofen synthesis, either the hydroformylation product **2** or the aminated derivative needs to be hydrolyzed. The methyl ester **1a** was chosen as substrate due to its atomic economy and its increased solubility compared to the ethyl and isopropyl ester. To theoretically enhance the regioselectivity by increasing the steric hindrance at the α‐position, we synthesized dimethyl 2‐(4‐chlorobenzylidene)malonate (**7**) in a Knoevenagel reaction with a good isolated yield of 51%. The hydroformylation of dimethyl 2‐(4‐chlorobenzylidene)malonate (**7**) resulted in predominantly formed hydrogenated by‐product **9** with 59–94% (**Scheme** **3**, for details see Supporting Information). Only <5% of aldehyde signal was obtained in ^1^H‐NMR spectra despite changing the gas composition of CO and H_2_ (from 6:1 to 1:3). Thus, dimethyl 2‐(4‐chlorobenzylidene)malonate (**7**) is not suitable as substrate for the regioselective hydroformylation required to synthesize β‐aldehyde **2**.

Regarding the applicability of hydroformylation and the space–time yield, we increased the substrate concentration stepwise from the standard conditions of 0.6 m following Botteghi and Paganelli^[^ ^15^ ^]^ and our previous work.^[^ ^13^ ^]^ Hydroformylation of methyl 4‐chlorochinnamate (**1a**) at 80 °C under 80 bar CO/H_2_ with substrate concentrations of 1.0–2.6 m resulted in high conversions of 91–97% (**Table** **2**).

**Table 2:** Hydroformylation of methyl 4‐chlorocinnamate (1a) with 1.0–2.6 m substrate concentration.

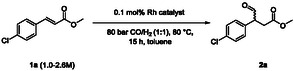				
Entry	Concentration [M]	Conversion [%]	Regioselectivity [%]a)	Selectivity [%]b)
1	1.0	91 ± 1	83 ± 2	65 ± 3
2	1.8	95 ± 1	84 ± 1	64 ± 2
3	2.6	97 ± 1	84 ± 1	63 ± 3

a) Reaction conditions: 1.0–2.6 m methyl 4‐chlorocinnamate (**1a**), “[HRh(PPh_3_)_4_]” (TCI, according to ICP‐OES: 0.1 mol% Rh), toluene, 80 bar CO/H_2_ (1:1), 80 °C, 15 h, *V*_total_ = 5 mL, double determination. Regioselectivity: % of β‐aldehyde **2** with respect to the sum of aldehydes **2** and **3**; determined by ^1^H‐NMR spectroscopy.

b) Selectivity: % β‐aldehyde **2** with respect to all detected by‐products (**4**, **5**, **6**) including the α‐aldehyde **3**.

Comparable regioselectivities of 83–84% and selectivities of 63–65% were obtained. In contrast to 0.6 m substrate concentration (88% regioselectivity, 68% selectivity),^[^ ^12^ ^]^ the regioselectivity and selectivity to the β‐aldehyde **2a** were slightly lower. With a substrate concentration of 2.6 m, the space–time yield was successfully increased by a factor of 4.3.

### 2.3 Ligand Screening

As reported recently by us, unmodified Rh complexes are the preferred catalytic species to obtain a high regioselectivity in hydroformylation of β‐aryl‐substituted α,β‐unsaturated esters, in comparison with the simple and widely used triphenylphosphine ligand.^[^ ^12^ ^]^ Based on these findings, we screened various different phosphine ligands compared to triphenylphosphine with the aim to investigate its electronic influence and to optimize the regioselectivity toward the β‐aldehyde **2a**. The hydroformylation of methyl 4‐chlorocinnamate (**1a**) was performed with 0.1 mol% [Rh(acac)(CO)_2_] and 0.5 mol% phosphine ligand under 80 bar CO/H_2_ (1:1) at 80 °C (**Table** **3**).

**Table 3:** Hydroformylation of methyl 4‐chlorocinnamate (1a) with 0.1 mol% [Rh(acac)(CO)_2_] and 0.5 mol% P‐ligand.

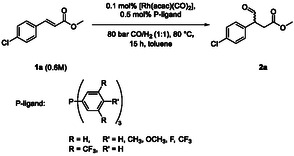					
**Entry**	***R***	***R*** **'**	**Conversion** **[%]**	**Regioselectivity** **[%]** a)	**Selectivity** **[%]** b)
1	H	OCH_3_ c)	77	37	24
2	H	CH_3_ c)	>99	40	22
3	H	Hd)	>99 ± 1	45 ± 5	28 ± 4
4	H	F	>99 ± 0	46 ± 2	26 ± 2
5	H	CF_3_	98 ± 0	60 ± 1	42 ± 1
6	CF_3_	He)	72 ± 8	74 ± 1	56 ± 3

a) Reaction conditions: 0.6 m (0.99 m e) methyl 4‐chlorocinnamate (**1a**), 0.1 mol% [Rh(acac)(CO)_2_] and 0.5 mol% P‐ligand, toluene, 80 bar CO/H_2_ (1:1), 80 °C, 15 h, *V*_total_ = 5 mL, reactions performed as duplicates. Regioselectivity: Amount of β‐aldehyde **2a** in relation to the sum of amounts of aldehyde **2a** and α‐aldehyde **3** (in %); determined by ^1^H‐NMR spectroscopy.

b) Selectivity: Amount of β‐aldehyde **2** in relation to all detected formed products (**2a**, **3a**, **4a**, **5a**, **6a**) (in %). By‐product **6a** was detected with ≤1%.

c) Single point determination.

d) This reaction served as our benchmark experiment and was recently published by us.^[^ ^12^ ^]^

e) 0.99 M substrate **1a**, 0.2 mol% [Rh(acac)(CO)_2_], 1 mol% P‐ligand.

According to the Tolman electronic parameters, tris(4‐methoxyphenyl)phosphine would be expected to have the most electron donating properties ($\tilde{v}$(CO) = 2066.1 cm^−1^),^[^ ^5^ ^]^ while the phosphine ligand is less electron‐rich with *R*' = CH_3_ $\tilde{v}$(CO) = 2066.7 cm^−1^),^[^ ^16^ ^]^ *H* ($\tilde{v}$(CO) = 2068.9 cm^−1^),^[^ ^5^ ^]^ and *F* ($\tilde{v}$(CO) = 2071.2 cm^−1^).^[^ ^16^ ^]^ The weakest donor properties are probably achieved with CF_3_, as this is a strongly electron withdrawing group.^[^ ^17^ ^]^ Corresponding to the assumed increasing electron withdrawing properties of the ligands, the regioselectivity was highest (74%) with the phosphine carrying two CF_3_ groups in *meta*‐position. With stronger electron donating groups as the methoxy group, only a regioselectivity of 37% was observed. The weaker coordinating tris[3,5‐bis(trifluormethyl)phenyl]phosphine compared to PPh_3_ could dissociate more easily and would probably generate HRh(CO)_4_.^[^ ^10^ ^]^ According to the literature, unmodified rhodium catalysts probably form rhodium‐carbonyl hydride [HRh(CO)_*x*_] (*x* = 3,4) as catalytic active species leading to the highest regioselectivity with methyl 4‐chlorocinnamate (**1a**) as substrate (see Table 1 and 2).

### 2.4 Scale‐Up and Product Isolation

The next step was focused on the product isolation. The work‐up of reaction mixtures after hydroformylation was processed in three different ways: filtration via celite including removal of the solvent, column chromatography, and the formation of the bisulfite adduct with subsequent release. The concept of bisulfite adduct formation is that aldehydes **2a** and **3a** are converted into water‐soluble bisulfite adducts, while substrate **1a** and by‐products remain in the organic phase. Afterward, the aldehyde is released. The easiest, most sustainable, and economic method is the first one. However, the formed by‐products cannot be separated with this method. Consequently, this work‐up can only be applied, if the following enzymatic transamination works in the presence of the most prevalent by‐product **4a**. For this reason, we compared the second and third method on a small scale (0.4–0.8 g crude product). After bisulfite adduct formation and release, as described in the literature for related aldehydes^[^ ^18^ ^]^ including an additional washing step, only an isolated yield of less than 2% was obtained. Purification by column chromatography resulted in a higher isolated yield of 32% for β‐aldehyde **2a** with a purity of 93% (7% α‐product **3a**). The efficacy of the hydroformylation of methyl 4‐chlorocinnamate (**1a**) was demonstrated through a scale‐up (fourfold increase) with 0.1 mol% [Rh(acac)(CO)_2_] as unmodified Rh complex. After 15 h, a high regioselectivity of 91% and a selectivity of 77% were achieved. Methyl 3‐(4‐chlorophenyl)‐4‐oxobutanoate (**2a**) was isolated, purified by column chromatography and obtained with a moderate yield of 41% and a purity of 92% (8% α‐aldehyde **3a**), thus indicating a slight increase of the regioisomeric ratio.

### 2.5 Preliminary Biocatalyst Screening

Amine transaminases are known for their superior stereoselectivity in the asymmetric conversion of ketones into (*S*)‐ or (*R*)‐amines.^[^ ^7^, ^19^ ^]^ However, many ATAs show only a moderate selectivity in the discrimination of the aldehyde's enantiomers. For the preparation of baclofen, an ATA had to be identified that converts the methyl ester of the precursor aldehyde, **2a**, or its hydrolyzed free acid, 3‐(4‐chlorophenyl)‐4‐oxobutanoic acid, **2d**. To identify suitable enzymes, we performed a two‐step screening procedure of our inhouse transaminase library, which contained 55 wild‐type ATAs and 200 variants.

The enzymes were produced by cultivating *E* *scherichia* *coli* BL21 cells harboring the coding ATA genes and subsequent purification of the proteins in 96‐well microtiter plates. In the first screening approach (Scheme S1A, Supporting Information), we conducted the reverse reaction and incubated *rac*‐Baclofen and *rac*‐Baclofen methyl ester as substrates with the enzymes together with pyruvate, as this is the universal amino acceptor used by most wild‐type ATA. The coproduct l‐alanine is then converted by alanine dehydrogenase to pyruvate and NADH, which then generates a colored formazan dye by subsequent steps (see Supporting Information). As (*R*)‐selective ATAs exclusively form d‐alanine as the coproduct, we included an alanine racemase in the assay mixture to expand the assay for screening ATAs with (*R*)‐selectivity. In the second screening reaction (Scheme S1B, Supporting Information), we employed the acetophenone assay to investigate enzymes in the desired forward reaction (see Supporting Information). In this approach, we used *rac*‐1‐phenylethaneamine as the amino donor with *rac*‐**2a** or **2d** as substrates. In case of activity, the coproduct acetophenone was detected at 245 nm. The advantage here is that enzymes that do not accept alanine as an amino donor can also be identified. From these screening reactions, we identified three wild‐type ATAs and 11 variants which showed activities in at least one of the assays. Especially interesting was to find a transaminase which also accepts isopropylamine as its amino donor.

After obtaining the screening results for potential enzymes for the conversion of the esterified aldehyde substrate, we decided to continue with the three most promising candidates from the screening, which showed the highest activity toward it. Thus, two variants of the transaminase from *Vibrio fluvialis* (56 V/57C/153 G/415C and 56 V/57C/415 F/417 A) and the wild‐type of the transaminase from *Arthrobacter* sp. KNK168 (ATA 117wt) were selected. All genes were expressed according to the standard expression method and the produced catalysts were used as lyophilized whole cells (dry weight whole cells—dwc) and/or lyophilized cell extracts. All enzyme variations showed activity with baclofen methyl ester substrate (methyl‐3‐(4‐chlorophenyl)‐4‐oxobutanoate) in test reactions (data not shown), whereas the typically (*R*)‐selective ATA 117wt proved to be the most promising candidate, displaying the highest activity with isopropylamine as its amino donor.^[^ ^20^ ^]^

### 2.6 Reaction Engineering toward Baclofen Synthesis Using the Transaminase ATA 117 from *Arthrobacter* sp. KNK168

Two rather similar substrates were chosen for the investigation: methyl‐4‐oxo‐3‐phenylbutanoate (**10**) and methyl‐3‐(4‐chlorophenyl)‐4‐oxobutanoate (**2a**, the final substrate for baclofen ester synthesis). **10**, being the nonhalogenated version of the actual baclofen substrate, was used for screenings to determine suitable reaction conditions. Furthermore, since during synthesis of both substrates 12–24% of a reduced by‐product (**4**) was formed, we investigated its potential to inhibit the ATA‐catalyzed reaction. All utilized substrates were synthesized via hydroformylation as described above and used in the subsequent biotransformations. The control reaction contained 100 mM of **10** and was incubated for 24 h with the reaction cocktail (1 mL; 50 mM HEPES pH8, 250 mM IPA, 2.5 mM PLP, 30 °C, 900 rpm, 80 U mL^−1^ of dwc with overexpressed ATA 117 wt). We observed the formation of 31 mM of product **11b**; however, in the presence of 50 mM chlorinated hydrogenated adduct (**4**), a significant inhibitory effect diminished the yield to only 14 mM of product. Thus, synthesized substrates would need to be purified prior to use.

**Scheme 4:**
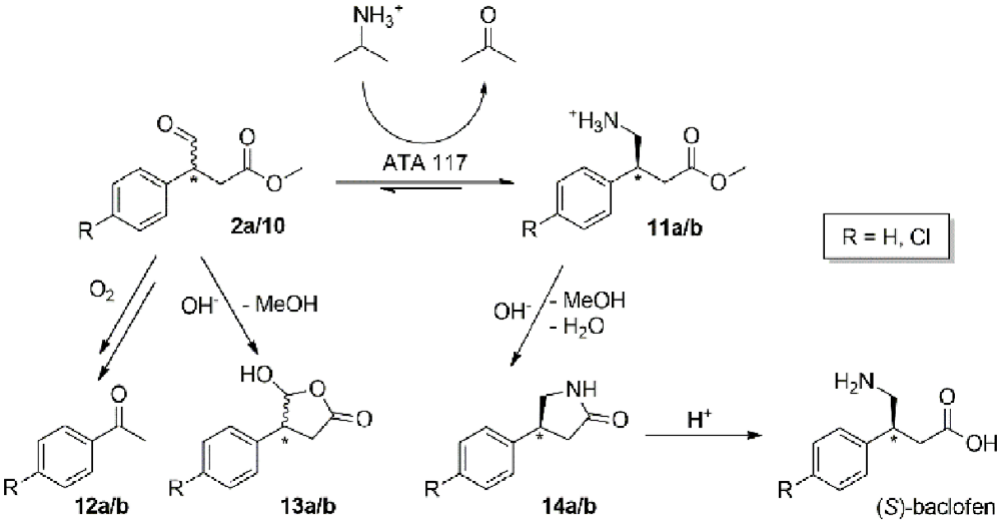
Overview of the baclofen ester synthesis with possible by‐products in basic environments. 2a: *R* = Cl, 10: *R* = H, 11/12/13/14a: *R* = Cl, 11/12/13/14b: *R* = H

To further shift the equilibrium of the baclofen synthesis, an in situ product removal approach was chosen. It was shown previously that baclofen esters tend to cyclize to the lactam 4‐(4‐chlorophenyl)‐2‐pyrrolidinone (**14**), also known as baclofen impurity A, under basic conditions (see **Scheme** **4**).^[^ ^21^ ^]^ The final product (*S*)‐baclofen is obtained from this intermediate via hydrolysis. Since transaminases usually work under basic conditions, some of the produced baclofen ester might cyclize and precipitate as a solid phase. If this fact could be exploited, it would yield higher conversions of baclofen from the last enzymatic synthetic step through a simple in situ product removal step (via a secondary solid phase) and thus shift the reaction equilibrium. However, two further by‐products were also detected in the reaction. As previously described for other α‐chiral aldehydes, O_2_ leads to the formation of acetophenone (**12**), which can be minimized by the degassing of the reaction solution previous to the start of the reaction.^[^ ^22^ ^]^ In addition, basic pH values lead to a minimal percentage of the substrate (**2a/10**) to cyclize into a lactone by‐product (**13**) (see Figure S4, Supporting Information).

**Figure 1:**
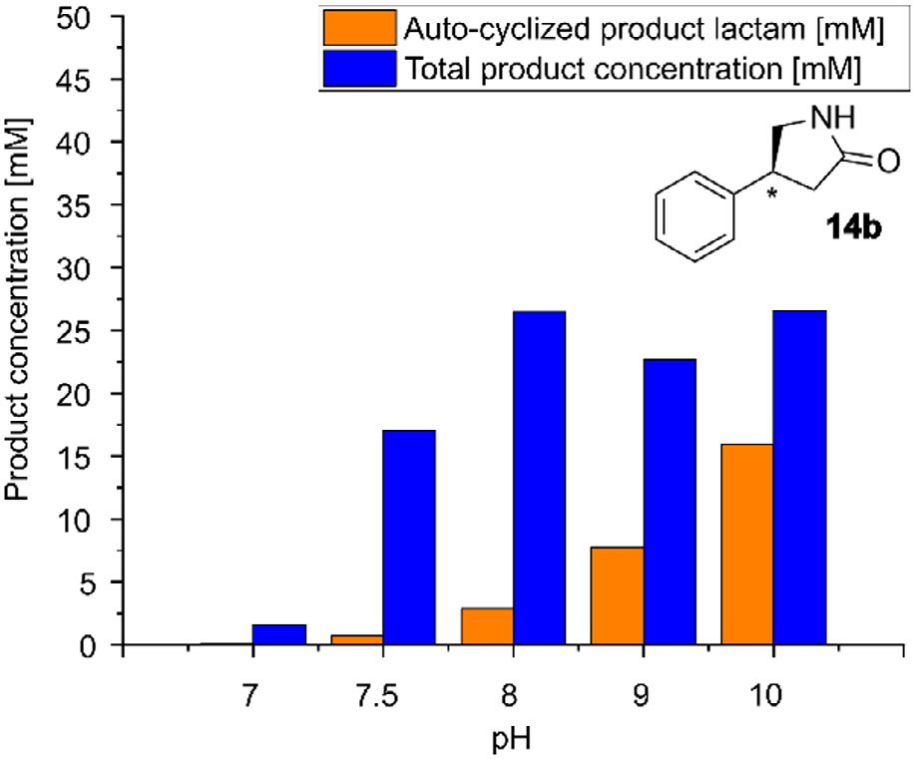
Influence of the pH on the conversion of **10** with ATA 117 wt and on the cyclization of the product to the lactam **14b**. Conditions: 50 mM HEPES buffer, 250 mM IPA, 2.5 mM PLP, 30 °C, 900 rpm, 24 h, 100 mM methyl‐4‐oxo‐3‐phenylbutanoate, 20 U mL^−1^ dwc, 1 mL.

Therefore, we investigated the influence of higher pH values on the reaction employing 100 mM of **10** at different pH values. This experiment revealed that the overall yield (sum of **11b** and **14b**) remained constant at a pH range of 8–10 (**Figure** **1**) despite rising pH values.

Although no inhibitory effect of the product lactamization on the enzyme was observed, no significant improvement of the overall yield (in situ product removal effect) was observed either, despite a higher ratio of autocyclization (see Figure 1). However, since having the product as a solid phase during the reaction was perceived as a downstream processing advantage, it was decided to continue with this line of work.

### 2.7 Conceptual Experiment for Baclofen Production

After this initial optimization with the nonchlorinated substrate analog, we went on to engineer the reaction by employing the true substrate **2a**. To increase product concentration, we designed the reactions as a fed‐batch, replenishing substrate consumption after 24 h. The substrate consumption was calculated as initial substrate concentration reduced by the formed concentrations of chloroacetophenone (**12a**), lactone by‐product (**13a**), and baclofen lactam (**14a**). After 24 h, an additional amount of the catalyst was added to compensate for the reduced enzyme activity due to deactivation (**Table** **4**). It turned out that the overall yield with the chlorinated substrate **2a** was higher than with the analog **10** (40 vs 30 mM product from 100 mM of substrate after 24 h). As expected, two factors influenced the yield of the produced baclofen. First, an increase of the pH value to 10 induced a slightly higher product formation than at pH 8 (Table 4), in contrast to the nonchlorinated substrate **10**, where no increase was observed with rising pH (Figure 1). Second, the addition of enzyme after 24 h at pH 10 facilitated a continued reaction, resulting in higher yields (48 mM compared to 75 mM). Otherwise, the reaction eventually stopped after 24 h. We hypothesize that the harsher conditions at pH 10 lead to an increased inactivation of the applied enzyme. Interestingly, while **2a** was successfully converted, no or negligible conversion of the regioisomer **3a** by the enzyme was observed (see Figure S5, Supporting Information).

**Table 4:** Production of baclofen methyl ester in fed‐batch reactions.

pH	Catalyst replenishment after [24 h]	Substrate replenishment after [24 h] [mM] (and total utilized substrate concentration [mM])	Total product (**14a**) concentration after 24 h [mM]	Total product (**14a**) concentration after 48 h [mM]
10	–	53.9 (total: 153.9)	41.1	48.0
8	20 U mL^−1^	37.9 (total: 137.9)	34.0	54.7
10	20 U mL^−1^	49.2 (total: 149.2)	39.4	74.7

Reaction conditions: 50 mM HEPES buffer, 250 mM IPA, 2.5 mM PLP, 30 °C, 900 rpm, 24 h, starting concentration of 100 mM methyl‐3‐(4‐chlorophenyl)‐4‐oxobutanoate (**2a**), 20 U mL^−1^ dwc, 1 mL. When catalyst was replenished, another 20 U mL^−1^ dwc were added to the mixture after 24 h. Substrate and IPA were replenished after 24 h.

Next, we isolated the reaction product **14a** of the best performing reaction (≈75 mM, pH 10, enzyme refilling) by extraction. The organic phase was separated and evaporated (evaporating residual extracted IPA as well), leaving only the extracted baclofen lactam with minor impurities of by‐products. The obtained solid was hydrolyzed in 2 mL of CPME containing 1.5 m HCl for 8 h at 100 °C. After evaporation, (*S*)‐baclofen was obtained with an enantiomeric ratio of 94:6 (*e.e.* 88%), as determined by chiral HPLC. The lowered stereoselectivity of the process could result either from slow substrate racemization or insufficient stereoselectivity of the enzyme. However, related studies and a conducted racemization NMR experiment (see Figure S11, Supporting Information) showed that the racemization of the aldehyde can be considered fairly fast, thus making the influence of kinetic effects on the stereoselectivity rather unlikely.^[^ ^23^ ^]^ This means that the stereoselectivity of the process is most probably lowered by effects of substrate positioning in the active site of the enzyme. For α‐chiral aldehyde substrates, an additional carbon atom separates the PLP‐bound region from the chiral center, disturbing substrate positioning and sometimes even leading to the inversion of the ω‐transaminase's stereoselectivity.^[^ ^22^ ^]^

## 3 Conclusion

In summary, this study successfully highlights a shortened chemoenzymatic synthetic route toward (*S*)‐baclofen, a viable compound for a number of applications, without the need for expensive catalysts or reagents. Conventionally utilized synthesis routes usually employ more synthetic steps and mostly produce racemic baclofen, lowering their atom efficiency due to the need for a subsequent chiral resolution step.^[^ ^1^, ^21^ ^]^ The concept involves a straight‐forward approach toward this exemplarily challenging β‐chiral amino acid, while using basically only one hydroformylation and one transaminase‐catalyzed reaction. Each individual synthesis step of the cascade process was thoroughly investigated; the observed side‐products identified almost without exception and minimized and the final synthesis executed to showcase its viability at synthetic scale. High conversions and a final product concentration of 75 mM with 88% *e.e.* (*S*) validated the developed synthetic approach, whereas especially the enantiomeric excess can be improved even further during subsequent downstream‐processing steps, e.g., enantioselective crystallization. Thus, future process development herein is expected to further improve overall performance and minimization of the undesired side products, incl. additional improvement to the (bio)catalysts itself, which was not part of this study.

## Conflict of Interest

The authors declare no conflict of interests.

## Supporting information

Supplementary Material

## Data Availability

The data that support the findings of this study are available in the supplementary material of this article.

## References

[1] A. Kleemann , J. Engel , B. Kutscher , D. Reichert , Pharmaceutical Substances, Georg Thieme Verlag, Stuttgart 2009.

[2] B. W. Cue , J. Zhang , Green Chem. Lett. Rev. 2009, 2, 193.

[3] a) M. E. Welsch , S. A. Snyder , B. R. Stockwell , Curr. Opin. Chem. Biol. 2010, 14, 347; 20303320 10.1016/j.cbpa.2010.02.018 PMC2908274

[4] A. J. Burke , C. S. Marques , N. J. Turner , G. J. Hermann , Active Pharmaceutical Ingredients in Synthesis, Wiley‐VCH, Weinheim, Germany 2018.

[5] P. Domínguez de María , Front. Catal. 2024, 3, 1359527.

[6] A. Liese , K. Seelbach , C. Wandrey (Eds.) Industrial Biotransformations, 2nd completely rev. and extended ed. ed., Wiley, Weinheim 2006.

[7] a) K. Drauz , H. Gröger , O. May , Enzyme Catalysis in Organic Synthesis, Wiley‐VCH, Weinheim, Germany 2012;

[8] a) Y. Cui , Y. Gao , L. Yang , Green Synth. Catal. 2024, https://10.1016/j.gresc.2024.03.003;

[9] a) J. W. Romito , E. R. Turner , J. A. Rosener , L. Coldiron , A. Udipi , L. Nohrn , J. Tausiani , B. T. Romito , SAGE Open Med. 2021, 9, 20503121211022197; 34158937 10.1177/20503121211022197 PMC8182184

[10] A. Börner , R. Franke , Hydroformylation. Fundamentals, Processes, and Applications in Organic Synthesis, Wiley, Weinheim 2016.

[11] R. Franke , D. Selent , A. Börner , Chem. Rev. 2012, 112, 5675. 22937803 10.1021/cr3001803

[12] H. Bork , T. Rösler , M. Leutzsch , N. Wessel , A. J. Vorholt , H. Gröger , Eur. J. Org. Chem. 2025, 28, e202401115.

[13] H. Bork , H. Gröger , Eur. J. Org. Chem. 2025, 28, e202401116.

[14] M. L. Clarke , G. J. Roff , Chem. Eur. J. 2006, 12, 7978. 16991187 10.1002/chem.200600914

[15] C. Botteghi , S. Paganelli , J. Organomet. Chem. 1993, 451, C18.

[16] C. A. Tolman , J. Am. Chem. Soc. 1970, 92, 2953.

[17] L. K. San , S. N. Spisak , C. Dubceac , S. H. M. Deng , I. V. Kuvychko , M. A. Petrukhina , X.‐B. Wang , A. A. Popov , S. H. Strauss , O. V. Boltalina , Chem. Eur. J. 2018, 24, 1441. 29178382 10.1002/chem.201704868

[18] a) X. Li , K. S. Iyer , R. R. Thakore , D. K. Leahy , J. D. Bailey , B. H. Lipshutz , Org. Lett. 2021, 23, 7205; 34472877 10.1021/acs.orglett.1c02604

[19] C. K. Savile , J. M. Janey , E. C. Mundorff , J. C. Moore , S. Tam , W. R. Jarvis , J. C. Colbeck , A. Krebber , F. J. Fleitz , J. Brands , P. N. Devine , G. W. Huisman , G. J. Hughes , Science 2010, 329, 305. 20558668 10.1126/science.1188934

[20] a) D. Koszelewski , D. Clay , D. Rozzell , W. Kroutil , Eur. J. Org. Chem. 2009, 2009, 2289;

[21] G. Veinberg , M. Vorona , A. Lebedevs , A. Chernobrovijs , I. Kalvinsh , WO2007096314A2 2007.

[22] a) F. Belov , A. Gazizova , H. Bork , H. Gröger , J. von Langermann , ChemBioChem 2024, 25, e202400203; 38602845 10.1002/cbic.202400203

[23] a) D. Koszelewski , D. Clay , K. Faber , W. Kroutil , J. Mol. Catal. B Enzym. 2009, 60, 191;

