# Supplementary material for: Chemoenzymatic Route toward a De Novo Enantioselective Total Synthesis of (S)‐Baclofen Based on Metal‐Catalyzed Hydroformylation and Enzymatic Transamination
Source: Chembiochem. 2025 Jul 4;26(15):e202500108. doi: 10.1002/cbic.202500108 (PMC12376269; doi:10.1002/cbic.202500108)
Supplement: Supplementary file 1 — Supplementary Material [file CBIC-26-e202500108-s001.pdf]

Supporting information

Chemoenzymatic route towards a de novo enantioselective total synthesis of (*S*)-baclofen based on metal-catalyzed hydroformylation and enzymatic transamination

Feodor Belov# [a], Hannah Bork# [b], Luise Hänel[c], Manideep V. Kollipara[c], Matthias Höhne* [c], Harald Gröger*[b] and Jan von Langermann*[a]

[a] M.Sc. Feodor Belov, Prof. Dr. Jan von Langermann*
Institute of Chemistry, Biocatalytic Synthesis Group
Otto von Guericke University of Magdeburg
Building 28, Universitätsplatz 2, 39106 Magdeburg (Germany)
E-mail: jan.langermann@ovgu.de

[b] M.Sc. Hannah Bork, Prof. Dr. Harald Gröger*
Faculty of Chemistry
Bielefeld University
Universitätsstrasse 25, 33615 Bielefeld (Germany)
Email: harald.groeger@uni-bielefeld.de

[c] M.Sc. Luise Hänel, M.Sc. Manideep Kollipara, Prof. Dr. Matthias Höhne
Institute of Chemistry
Technical University of Berlin
Müller-Breslau-Strasse 10, 10623 Berlin (Germany)
Email: matthias.hoehne@tu-berlin.de

#both authors share first authorship

Table of contents

1. MATERIAL AND METHODS 3

2. EXPERIMENTAL SECTION 4

2.1 Synthesis of substrates 4

2.2 Synthesis of cinnamates and reference compounds 5

2.3 Hydroformylation 7
Hydroformylation of methyl 4-chlorocinnamate (1a) 7
Hydroformylation of ethyl 4-chlorocinnamate (1b) 8
Hydroformylation of isoproyl 4-chlorocinnamate (1c) 8
Hydroformylation of 2-(4-chlorobenzylidene)malonate (7) 9

2.4 Isolation of product and scale-up 10

2.5 Enzyme Screening 12

2.6 Preparation of biocatalysts 14

2.7 Biocatalytic reactions 16

2.8 Analysis of biocatalytic reaction products 16

2.9 NMR spectra 18

2.10 GC and HPLC chromatograGC 23

2.11 IR spectra 26

2.12 Racemization study for the substrate 2a 27

2.13 References 28

1. Material and Methods

Chemicals

All required chemicals were commercially available and purchased from BldPharm, Sigma-Aldrich, Merck, Strem, Acros Organics, VWR, Abcr, Carbolution, Alfa Aesar, Thermo Fisher scientific, Fluka, Deutero and Eurisotop and were used without further purification. The Rh-catalyst named $[HRh(PPh_3)_4]$ was obtained from TCI (CAS: 18284-36-1, Lot-Nr.: P6NQM-ED). 4-Chlorocinnamic acid was purchased from Fluorochem. Racemic baclofen was purchased from TCI Chemicals, enantiopure (*R*)-baclofen was purchased from abcr. Synthesis gas was used premixed from Messer (CO ≥99.997%, H_2 ≥99.999%) or added separately (CO: Messer, ≥99.997% and H_2: Linde, ≥99.999%).

Nuclear magnetic resonance (NMR) spectroscopy

All reactions were analyzed by NMR spectroscopy. ^{1}H NMR, $^{13}C\{^{1}H\}$ and 2D NMR spectra were recorded using a Bruker Avance III 400, Bruker Avance III 500 HD, Bruker Avance III 500 or Bruker Avance 600 instrument. Unless otherwise noted, measurements were performed at 298 K. Chemical shifts δ are reported in ppm and the residual hydrogen signals of the solvents were used as reference. The multiplicity of the signals is given in brackets and is abbreviated as follows: s = singlet, d = doublet, t = triplet and m = multiplet. The coupling constant J refer to $^{1}H,^{1}H$ couplings is given in Hertz (Hz). The conversion, regioselectivity and selectivity were determined from the ^{1}H-NMR spectra according to our recent publication.[26]

Accurate mass (ESI)

Nano-ESI measurements were performed using a Q-IMS-TOF mass spectrometer Synapt G2Si (Waters GmbH, Manchester, UK) in resolution mode, interfaced to a nano-ESI ion source. Nitrogen serves as the nebulizer gas and the dry gas for nano-ESI. Nitrogen was generated by a nitrogen generator NGM 11. Samples were dissolved in acetonitrile or acetonitrile/dichloromethane and introduced by static nano-ESI using in-house pulled glass emitters. The mass axis was externally calibrated using ESI-L Low Concentration Tuning Mix (Agilent Technologies, Santa Clara, USA) and each measurement was internally calibrated with the protonated LeuEnk ion as internal calibration standard. Scan accumulation and data processing was performed with MassLynx 4.1 (Waters GmbH, Manchester, UK) on a PC Workstation. The data shown here were generated by the accumulation and averaging of several single spectra (scan time 1 minute, 58 scans). Determination of exact mass was performed using centroided data.

CHN analysis

For CHN analysis, the EURO EA Elemental Analyzer from Eurovector was used.

Infrared (IR) spectroscopy

IR spectra were measured with a Thermo Nicolet 380 or iS20 FTIR spectrometer from Thermo Fisher Scientific.

Gas chromatography coupled to mass spectrometry (GC-MS)

The qualitative analysis of products was determined by a *Shimadzu* Nexis GC-2030 with a *Phenomenex* Zebron ZB-5MSi (0.20 mm ID, 0.25 µm film thickness and 30 m length) column and a QP mass detector (for exemplary visualized data see Figure S9).
Settings split: 300 °C, carrier gas: He, pressure: 155.3 kPa, total flow: 21.6 mL/min, column flow: 0.89 mL/min, linear velocity: 35.0 cm/s, purge flow: 3.0 mL/min, split ratio: 20.0.
Settings oven: 100 to 330 °C with 20 °C/min, 300 °C for 5.30 min.
Settings QP2020: ion source temperature: 240 °C, interface temperature: 350 °C, time: 3.10 min-16.00 min, from 35.00 to 800.0 *m/z*.

Purification by column chromatography
The column chromatographic purification was performed using the *Biotage Isolera One* with *Biotage* Sfaer Silica HC D or SNAP Ultra columns as stationary phase and cyclohexane/ethyl acetate as solvent.

Hydroformylation
All hydroformylations were performed in high pressure autoclaves (75 mL from Parr) of a multi reactor system equipped with glass or teflon inlets and stirring bars. The conversion, regioselectivity and selectivity were determined by ^{1}H NMR spectroscopy. Hydroformylations of 2-(4-chlorobenzylidene)malonate (**7**) with varied CO/H_2 composition were performed in self-made autoclaves (10-20 mL) at MPI CEC in Mülheim.

2. Experimental Section

2.1 Synthesis of substrates

Synthesis of methyl 4-chlorocinnamate (**1a**)
Methanol (320 mL) and concentrated sulfuric acid (16 mL) were added to cinnamic acid (16.0 g, 87.6 mmol, 0.27 M). The reaction mixture was heated to reflux for 17 h and cooled to room temperature afterwards. Subsequently, one half to two thirds of the solvent were removed and dH_2O (160 mL) was added. After extraction with ethyl acetate (1 x 300 mL, 2 x 250 mL), the combined organic layers were washed with sodium hydrogencarbonate solution (5 % *w/w* in dH_2O, 1 x 300 mL) and dH_2O (3 x 350 mL). The resulting organic layer was dried over magnesium sulfate and the solvent was evaporated. Methyl 4-chlorocinnamate (**1a**) was obtained as colorless solid (15.3 g, 77.8 mmol, 89% yield). The spectroscopic data is in accordance with the literature.[1]

^{1}H-NMR (500 MHz, $CDCl_3$): δ [ppm] = 7.64 (d, J = 15.9 Hz, 1H, *H*C=C), 7.45 (m, 2H, C_6H_4Cl), 7.36 (m, 2H, C_6H_4Cl), 6.41 (d, J = 15.9 Hz, 1H, *H*C=C), 3.81 (s, 3H, CH_3).
^{13}C{^{1}H}-NMR (126 MHz, $CDCl_3$): δ [ppm] = 167.3 (1C, *C*O), 143.6 (1C, *C*H), 136.4 (1C, qC_6H_4Cl), 133.0 (1C, qC_6H_4Cl), 129.4 (2C, C_6H_4Cl), 129.3 (2C, C_6H_4Cl), 118.5 (1C, *C*H), 51.9 (1C, CH_3).

Synthesis of ethyl 4-chlorocinnamate (**1b**)
Ethanol (200 mL) and concentrated sulfuric acid (2 mL) were added to cinnamic acid (1.00 g, 5.48 mmol, 27 mM) and the reaction mixture was heated to reflux for 18 h. Afterwards, two thirds of the solvent were removed and dH_2O (100 mL) was added. After extraction with ethyl acetate (3 x 200 mL), the combined organic layers were washed with sodium hydrogencarbonate solution (5 % *w/w* in dH_2O, 1 x 250 mL) and dH_2O (2 x 250 mL). The organic layer was dried over magnesium sulfate. The evaporation of the solvent yielded ethyl 4-chlorocinnamate (**1b**, 1.05 g, 4.98 mmol, 91% yield) as colorless oil. The spectroscopic data is in accordance with the literature.[1]

^{1}H-NMR (500 MHz, $CDCl_3$): δ [ppm] = 7.62 (d, J = 15.9 Hz, 1H, *H*C=C), 7.44 (m, 2H, C_6H_4Cl), 7.35 (m, 2H, C_6H_4Cl), 6.40 (d, J = 16.1 Hz, 1H, *H*C=C), 4.25 (m, 2H, CH_2), 1.32 (m, 3H, CH_3).
Accurate mass (ESI): $[C_{11}H_{11}ClO_2 + Na]^+$ *m/z* (calcd): 233.03398, *m/z* (found): 233.0345.

Synthesis of isopropyl 4-chlorocinnamate (**1c**)
Isopropanol (80 mL) and concentrated sulfuric acid (5 mL) were added to cinnamic acid (4.02 g, 22.0 mmol, 0.28 M). The reaction mixture was heated to reflux for 13 h and cooled to room temperature. Afterwards, one half to two thirds of the solvent were removed and dH_2O (40 mL) was added. After extraction with ethyl acetate (1 x 70 mL), the combined organic

layers were washed with sodium hydrogencarbonate solution (5 % *w/w* in dH_2O, 1 x 70 mL) and dH_2O (1 x 100 mL). Due to poor phase separation, saturated NaCl solution was added and the organic phase was washed again with dH_2O (3 x 100 mL). The organic layer was dried over magnesium sulfate and the solvent was evaporated. The resulting crude product (4.05 g) was purified by column chromatography (1 CV 5% EtOAc, 10 CV 5-40% EtOAc, 2 CV 40% EtOAc, 100 mL/min, 100 g SNAP Ultra column). Isopropyl 4-chlorocinnamate (**1c**) was obtained as colorless solid (1.47 g, 6.54 mmol, 30% yield). The spectroscopic data is in accordance with the literature.[1]

^{1}H-NMR (500 MHz, DMSO-d_6): δ [ppm] = 7.61 (d, J = 16.0 Hz, 1H, *H*C=C), 7.44 (m, 2H, C_6H_4Cl), 7.35 (m, 2H, $C_6\mathit{H}_4Cl$), 6.38 (d, J = 16.1 Hz, 1H, *H*C=C), 5.14 (m, 1H, *H*C(CH_3)(CH_3)), 1.31 (d, J = 6.2 Hz, 6H, $C\mathit{H}_3$).
Accurate mass (ESI): $[C_{12}H_{13}ClO_2 + Na]^+$ *m/z* (calcd): 247.04963, *m/z* (found): 247.0494.

<u>Synthesis of dimethyl 2-(4-chlorobenzylidene)malonate (**7**)</u>
According to literature[2], 4-chlorobenzaldehyde (2.66 g, 18.9 mmol, 1.00 eq) was introduced with toluene (35 mL), and dimethyl propanedioate (2.47 g, 18.7 mmol, 0.99 eq.) and piperidine (0.40 mL, 4.0 mmol, 0.21 eq.) were added. After heating to reflux for 63 h, dH_2O (20 mL) was added, and the pH of the aqueous phase was adjusted to 5. The organic phase was washed with dH_2O (2 x 30 mL), and the last aqueous phase was extracted with toluene (60 mL). The organic phases were combined and dried over $MgSO_4$. The solvent was removed and the crude product (4.24 g) was purified by column chromatography (1 CV 0% EtOAc, 20 CV 0-4% EtOAc, 9.6 CV 4% EtOAc, 120 mL/min, 100 g Sfaer Silica HC D column). Dimethyl 2-(4-chlorobenzylidene)malonate (**7**) was obtained as a slightly yellowish solid (2.44 g, 9.58 mmol, 51% yield). The spectroscopic data is in accordance with the literature.[3]

^{1}H-NMR (500 MHz, $CDCl_3$): δ [ppm] = 7.72 (s, 1H, *H*C=C), 7.36 (s, 4H, $C_6\mathit{H}_4Cl$), 3.85 (s, 3H, $C\mathit{H}_3$), 3.84 (s, 3H, $C\mathit{H}_3$).
^{13}C-NMR (126 MHz, $CDCl_3$): δ [ppm] = 166.9 (*C*O_2CH_3), 164.4 (*C*O_2CH_3), 141.6 (H*C*=C), 136.9 (*C*(CO_2CH_3)$_2$),131.4 ($C_6\mathit{H}_4Cl$), 130.7 ($C_6\mathit{H}_4Cl$), 129.3 ($C_6\mathit{H}_4Cl$), 126.2 ($C_6\mathit{H}_4Cl$), 52.9 (2 C, *C*H_3).
Accurate mass (ESI): $[C_{12}H_{11}ClO_4 + Na]^+$ *m/z* (calcd): 277.02381, *m/z* (found): 277.024.

2.2 Synthesis of cinnamates and reference compounds

<u>Synthesis of methyl 4-oxo-3-phenylbutanoate (**10**)</u>
The synthesis of methyl 4-oxo-3-phenylbutanoate was prepared in a Dean-Stark apparatus under inert conditions until the reaction was stirred at room temperature with acetic acid and dH_2O. 2-phenylacetaldehyde (50.4 g, 49.1 mL, 419 mmol, 1.00 eq.) was added dropwise to diisobutylamine (54.4 g, 73.5.0 mL, 421 mmol, 1.00 eq.) in dry toluene (275 mL) over 30 min. The mixture was heated to reflux for 2 h and the solvent was removed under reduced pressure. The day after, the residue was dissolved in dry acetonitrile (70 mL) and a solution of methyl bromoacetate (64.0 g, 39 mL, 0.42 mol, 1.00 eq.) in dry acetonitrile (40 mL) was added dropwise over 30 min. The mixture was heated to reflux for 4 h and the day after, acetic acid (26 mL) and dH_2O (75 mL) were added. The mixture was stirred for 30 min at room temperature and heated to 45 °C for 1 h. The solvent was removed under reduced pressure and water (200 mL) was added afterwards. Subsequently the aqueous phase was extracted with dichloromethane (2 x 300 mL, 1 x 250 mL), the combined organic phase was washed with saturated $NaHCO_3$ solution (2 x 100 mL) and dried over magnesium sulfate. By removing the solvent under reduced pressure, the crude product was obtained. Purification by fractional vacuum distillation (0.011 mbar, 170 °C) yielded a yellow oil (42.3 g). 5.10 g of the yellow oil were further purified by column filtration over silica (*n*-hexane/ethylacetate (8:2)). After evaporation of the solvent, methyl 4-oxo-3-phenylbutanoate was obtained as yellow oil (4.01 g). The spectroscopic data (Fig. S1) are in accordance to Komnatnyy *et al.*[4]

^{1}H-NMR (500 MHz, $CDCl_3$): δ [ppm] = 9.70 (s, 1H, C*H*O), 7.39 (m, 2H, C_6H_5), 7.33 (m, 1H, C_6H_5), 7.20 (m, 2H, C_6H_5), 4.16 (dd, J = 8.4, 6.1 Hz, 1H, C*H*CHO), 3.67 (s, 3H, CH_3), 3.17 (dd, J = 16.8, 8.4 Hz, 1H, C*H*), 2.62 (dd, J = 16.8, 6.1 Hz, 1 H, C*H*).
^{13}C{^{1}H}-NMR (126 MHz, $CDCl_3$): δ [ppm] = 198.7, (1C, *C*HO), 172.2 (1C, *C*O), 134.9 (1C, C_6H_5), 129.5 (1C, C_6H_5), 129.0 (2C, C_6H_5), 128.2 (2C, C_6H_5), 54.8 (1C, *C*HCHO), 52.1 (1C, CH_3), 34.5 (1C, CH_2).

Synthesis of methyl 3-(4-chlorophenyl)propanoate (**4a**)

Methanol (99 mL) and concentrated sulfuric acid (1.5 mL) were added to 3-(4-chlorophenyl)-propanoic acid (1.45 g, 7.85 mmol, 79 mM). The reaction mixture was stirred for 22 h at room temperature. Afterwards, half of the solvent was removed and dH_2O (40 mL) was added. After extraction with ethyl acetate (3 x 50 mL), the combined organic layers were washed with sodium hydrogencarbonate solution (5 % *w/w* in dH_2O, 1 x 50 mL) and dH_2O (3 x 100 mL). The organic layer was dried over magnesium sulfate and the solvent was evaporated. Methyl 3-(4-chlorophenyl)propanoate (**4a**) was obtained as colorless oil (1.13 g, 5.69 mmol, 72% yield). The spectroscopic data is in accordance with the literature.[5]

^{1}H-NMR (500 MHz, $CDCl_3$): δ [ppm] = 7.25 (m, 2H, C_6H_4Cl), 7.13 (m, 2H, C_6H_4Cl), 3.67 (s, 3H, CH_3), 2.92 (t, J = 7.7 Hz, 2H, H_2C-C_6H_4Cl), 2.61 (t, J = 7.7 Hz, 2H, H_2C-CO_2CH_3).
^{1}H-NMR (500 MHz, DMSO-d_6): δ [ppm] = 7.32 (m, 2H, C_6H_4Cl), 7.25 (m, 2H, C_6H_4Cl), 3.57 (s, 3H, CH_3), 2.83 (t, J = 7.6 Hz, 2H, H_2C-C_6H_4Cl), 2.62 (t, J = 7.6 Hz, 2H, H_2C-CO_2CH_3).
Accurate mass (ESI): $[C_{10}H_{11}ClO_2 + Na]^+$ *m/z* (calcd): 221.03398, *m/z* (found): 221.0338.

Synthesis of ethyl 3-(4-chlorophenyl)propanoate (**4b**)

The synthesis of ethyl 3-(4-chlorophenyl)propanoate was performed according to the synthesis of methyl 3-(4-chlorophenyl)propanoate (**4**).[5] Ethanol (14 mL) and concentrated sulfuric acid (0.2 mL) were added to 3-(4-chlorophenyl)propanoic acid (0.20 g, 1.1 mmol, 14 mM). The reaction mixture was stirred at room temperature and after 21 h, half of the solvent was removed. dH_2O (5 mL) was added and the aqueous phase was extracted with ethyl acetate (3 x 10 mL). After the combined organic layers were washed with sodium hydrogencarbonate solution (5 % *w/w* in dH_2O, 1 x 5 mL) and dH_2O (2 x 5 mL), the organic layer was dried over magnesium sulfate. Evaporation of the solvent resulted in ethyl 3-(4-chlorophenyl)propanoate (**4b**) as colorless oil (0.11 g, 0.52 mmol, 48% yield). The spectroscopic data is in accordance with the literature.[6]

^{1}H-NMR (500 MHz, DMSO-d_6): δ [ppm] = 7.33 (m, 2H, C_6H_4Cl), 7.26 (m, 2H, C_6H_4Cl), 4.04 (q, J = 7.1 Hz, 2H, CH_2-CH_3), 2.84 (t, J = 7.5 Hz, 2H, H_2C-C_6H_4Cl), 2.61 (t, J = 7.6 Hz, 2H, H_2C-CO_2CH_2), 1.15 (t, J = 7.1 Hz, 3H, CH_3).

Synthesis of isopropyl 3-(4-chlorophenyl)propanoate (**4c**)

The synthesis of isopropyl 3-(4-chlorophenyl)propanoate was performed according to the synthesis of methyl 3-(4-chlorophenyl)propanoate (**4**).[5] Isopropanol (14 mL) and concentrated sulfuric acid (0.2 mL) were added to 3-(4-chlorophenyl)propanoic acid (0.20 g, 1.1 mmol, 14 mM). The reaction mixture was stirred for 23 h at room temperature. Afterwards, half of the solvent was removed and dH_2O (5 mL) was added. After extraction with ethyl acetate (3 x 10 mL), the combined organic layers were washed with sodium hydrogencarbonate solution (5 % *w/w* in dH_2O, 1 x 5 mL) and dH_2O (2 x 5 mL). The resulting organic layer was dried over magnesium sulfate and the solvent was evaporated. The crude product was purified by column chromatography (1 CV 5% EtOAc, 20 CV 5-40% EtOAc, 2 CV 40% EtOAc, 18 mL/min Sfaer Silica 5 g HC D column) and isopropyl 3-(4-chlorophenyl)-propanoate (**4c**) was obtained as colorless oil (46 mg, 0.20 mmol, 20% yield). The spectroscopic data is in accordance with the literature for isopropyl 3-phenylpropanoate.[7]

^{1}H-NMR (500 MHz, $CDCl_3$): δ [ppm] = 7.27 (m, 2H, C_6H_4Cl), 7.16 (m, 2H, C_6H_4Cl), 5.01 (m, 1H, CO_2CH), 2.93 (t, J = 7.7 Hz, 2H, H_2C-C_6H_4Cl), 2.59 (t, J = 7.7 Hz, 2H, H_2C-CO_2CH), 1.22 (d, J = 6.3 Hz, 6H, CH_3).
^{13}C{^{1}H}-HMR (126 MHz, $CDCl_3$): δ [ppm] = 172.3 (1C, CO_2CH), 139.2 (1C, qC_6H_4Cl), 132.1 (1C, qC_6H_4Cl), 129.8 (2C, C_6H_4Cl), 128.7 (2C, C_6H_4Cl),) 68.0 (1C, CH), 36.2 (1C, H_2C-CO_2CH), 30.5 (1C, H_2C-C_6H_4Cl), 21.9 (2C, CH_3).
CHN: calculated C: 63.58, H: 6.67, found: C: 63.72, H: 6.96.
Accurate mass (ESI): $[C_{12}H_{15}ClO_2 + Na]^+$ *m/z* (calcd): 249.06528, *m/z* (found): 249.0665.
IR (neat): $\tilde{\nu}$ [cm^{-1}] = 1728 ($C{=}O_{ester}$).

2.3 Hydroformylation

Unless otherwise noted, hydroformylations were performed in double determination.

Hydroformylation of methyl 4-chlorocinnamate (**1a**)
For the hydroformylation of methyl 4-chlorocinnamate (**1a**) with 0.20 M substrate concentration, methyl 4-chlorocinnamate (**1a**, 0.20 g, 1.0 mmol, 0.20 M) and "$[HRh(PPh_3)_4]$" (1.1 mg, according to ICP-OES:[8] 0.095 mol% Rh) were weighed in the glass inlet which was subsequently inserted into the reactor. The autoclave was then purged with argon. Under an argon atmosphere, dry toluene (5 mL) was added. The autoclave was inserted in the multi reactor system, stirred at 1000 rpm and purged with nitrogen (3x20-30 bar) and hydrogen (3x20-30 bar) or syngas (CO/H_2 (1:1), 3x20-30 bar). Then it was pressurized with 80 bar CO/H_2 (1:1) and heated to 80 °C for 15 h. Afterwards, the reactor was depressurized and purged with nitrogen (3x20-30 bar) again. From a sample of the reaction solution, the solvent was evaporated.
The hydroformylation of methyl 4-chlorocinnamate (**1a**) with higher substrate concentration (1.0-2.6 M) was performed analogous to the 0.20 M reaction. For 1.0 M reaction, substrate **1a** (0.98 g, 5.0 mmol, 1.0 M) and "$[HRh(PPh_3)_4]$" (5.9 mg, according to ICP-OES:[8] 0.10 mol% Rh) were added, for 1.8 M substrate concentration, substrate **1a** (1.78 g, 9.06 mmol, 1.8 M) and "$[HRh(PPh_3)_4]$" (11 mg, according to ICP-OES:[8] 0.10 mol% Rh) were used. In case of the 2.6 M substrate, methyl 4-chlorocinnamate (**1a**, 2.56 g, 13.0 mmol, 2.6 M) and "$[HRh(PPh_3)_4]$" (15 mg, according to ICP-OES:[8] 0.098 mol% Rh) were added. The conversion, regioselectivity and selectivity for all reactions were determined by ^{1}H NMR spectroscopy and are shown in Table S1. The amounts of formed products in percentage are present in Table S2.

Table S1: Hydroformylation of methyl 4-chlorocinnamate (**1a**) with 0.2 to 2.6 M substrate concentration. As result, the conversion, regioselectivity and selectivity is shown.

Substrate concentration	Conversion [%]	Regioselectivity [%]	Selectivity [%]
0.2 M	78 ± 6	89 ± 2	68 ± 1
1.0 M	91 ± 1	83 ± 2	65 ± 3
1.8 M	95 ± 1	84 ± 1	64 ± 2
2.6 M	97 ± 1	84 ± 1	63 ± 3

Table S2: Hydroformylation of methyl 4-chlorocinnamate (**1a**) with 0.2 to 2.6 M substrate concentration. As result, the percentage of all detected products are shown. α-aldehyde **3a** includes the tautomeric α-enol.

Substrate concentration	2a [%]	3a [%]	4a [%]	5 [%]	6a [%]
0.2 M	53 ± 3	6 ± 2	15 ± 1	< 1	2 ± 0.2
1.0 M	60 ± 1	12 ± 1	19 ± 2	< 1	< 1
1.8 M	62 ± 1	12 ± 1	21 ± 2	< 1	< 1
2.6 M	62 ± 2	12 ± 0.3	22 ± 2	< 1	< 1

Hydroformylation of ethyl 4-chlorocinnamate (**1b**)

Ethyl 4-chlorocinnamate (**1b**, 0.21 g, 1.0 mmol, 0.20 M) and "[$HRh(PPh_3)_4$]" (1.3 mg, according to ICP-OES:[8] 0.14 mol% Rh) were weighed in the glass inlet which was subsequently inserted into the reactor. The autoclave was then purged with argon. Under an argon atmosphere, dry toluene (5 mL) was added. The autoclave was inserted in the multi reactor system, stirred at 1000 rpm and purged with nitrogen (3x20-30 bar) and hydrogen (3x20-30 bar) or syngas (CO/H_2 (1:1), 3x20-30 bar). Then it was pressurized with 80 bar CO/H_2 (1:1) and heated to 80 °C for 15 h. Afterwards, the reactor was depressurized and purged with nitrogen (3x20-30 bar) again. From a sample of the reaction solution, the solvent was evaporated. According to ^{1}H-NMR spectroscopy, 56 ± 8% of β-aldehyde **2b**, 5 ± 1% of α-product **3b**, 17 ± 4% of by-product **4b** and < 1% of by-product **5** were detected. A conversion of 79 ± 12%, a regioselectivity of 91 ± 1% and a selectivity of 71 ± 1% was obtained. The solvent was removed from reaction solution and the crude product was purified by column chromatography (1 CV 5% EtOAc, 35 CV 5-40% EtOAc, 2 CV 40% EtOAc, 18 mL/min Sfaer Silica 5 g HC D column) yielding β-aldehyde **2b** (7 mg, 0.03 mmol, 3% yield).
^{1}H-NMR (500 MHz, CDl_3): δ [ppm] = 9.68 (s, 1H, C*H*O), 7.35 (m, 2H, C_6H_4Cl), 7.14 (m, 2H, C_6H_4Cl), 4.12 (m, 3H, C*H*CHO, CH_2CH_3), 3.13 (dd, J = 16.9, 8.0 Hz, 1 H, C*H*), 2.59 (dd, J = 16.8, 6.5 Hz, 1 H, C*H*), 1.22 (t, J = 7.1 Hz, 3H, CH_3).
^{13}C{^{1}H}-HMR (126 MHz, $CDCl_3$): δ [ppm] = 198.2, (1C, *C*HO), 171.4 (1C, *C*O), 134.3 (1C, qC_6H_4Cl), 133.4 (1C, qC_6H_4Cl), 130.4 (2C, C_6H_4Cl), 129.6 (2C, C_6H_4Cl), 61.1 (1C, CH_2CH_3), 54.1 (1C, *C*HCHO), 34.8 (1C, CH_2), 14.3 (CH_3).
Accurate mass (ESI): [$C_{12}H_{13}ClO_3$ + Na]$^+$ *m/z* (calcd): 263.04454, *m/z* (found): 263.0454.
GC-MS (EI): 51.1, 77.1, 89.1, 103.1, 125.1, 138.1, 140.1, 167.0, 195.0, 198.1, 212.1.
IR (neat): $\tilde{\nu}$ [cm^{-1}] = 1736-1720 ($C{=}O_{ester}$, $C{=}O_{aldehyde}$).

Hydroformylation of isoproyl 4-chlorocinnamate (**1c**)

Isopropyl 4-chlorocinnamate (**1c**, 0.22 g, 1.0 mmol, 0.20 M) and "[$HRh(PPh_3)_4$]" (1.2 mg, according to ICP-OES:[8] 0.10 mol% Rh) were weighed in the glass inlet which was subsequently inserted into the reactor. The autoclave was purged with argon and under an argon atmosphere, dry toluene (5 mL) was added. After inserting the autoclave in the multi reactor system, it was stirred at 1000 rpm and purged with nitrogen (3x20-30 bar) and hydrogen (3x20-30 bar) or syngas (CO/H_2 (1:1), 3x20-30 bar). Then the autoclave was pressurized with 80 bar CO/H_2 (1:1) and heated to 80 °C for 15 h. Afterwards, the reactor was depressurized and purged with nitrogen (3x20-30 bar). From a sample of the reaction solution, the solvent was evaporated. According to ^{1}H-NMR, 68 ± 0.1% of β-aldehyde **2c**, 7 ± 1% of α-product **3c**, 16 ± 2% of by-product **4c** and < 1% of by-product **5** were detected. A conversion of 91% ± 1, a regioselectivity of 91 ± 1% and a selectivity of 75 ± 1% was determined. The solvent was removed from reaction solution and the crude product was purified by column chromatography (1 CV 5% EtOAc, 20 CV 5-40% EtOAc, 2 CV 40% EtOAc, 18 mL/min Sfaer Silica 5 g HC D column) yielding β-aldehyde **2c** (55 mg, 0.22 mmol, 22% yield).

^{1}H-NMR (500 MHz, $CDCl_6$): δ [ppm] = 9.68 (s, 1H, C*H*O), 7.36 (m, 2H, C_6*H_4*Cl), 7.14 (m, 2H, C_6*H_4*Cl), 4.97 (m, 1H, C*H*(CH_3)$_2$), 4.12 (m, 1H, C*H*CHO), 3.09 (dd, J = 16.6, 8.0 Hz, 1 H, C*H*), 2.57 (dd, J = 16.6, 6.7 Hz, 1 H, C*H*), 1.21 (d, 6.3 Hz, 3H, C*H_3*), 1.16 (d, 6.3 Hz, 3H, C*H_3*).
^{13}C{^{1}H}-HMR (126 MHz, $CDCl_3$): δ [ppm] = 198.2, (1C, *C*HO), 170.9 (1C, *C*O), 134.3 (1C, q*C_6*H_4Cl), 133.5 (1C, q*C_6*H_4Cl), 130.4 (2C, *C_6*H_4Cl), 129.6 (2C, *C_6*H_4Cl), 68.6 (1C, *C*H(CH_3)$_2$), 54.2 (1C, *C*HCHO), 35.1 (1C, *C*H_2), 21.9 (2C, *C*H_3).
CHN: calculated C: 61.30, H: 5.94, found: C: 61.41, H: 6.15.
Accurate mass (ESI): [$C_{13}H_{15}ClO_3$ + Na]$^+$ *m/z* (calcd): 277.06019, *m/z* (found): 277.0610.
IR (neat): $\tilde{\nu}$ [cm^{-1}] = 1734-1720 (C=O$_{ester}$, C=O$_{aldehyde}$).
198.1, (1C, *C*HO), 171.9 (1C, *C*O), 134.4 (1C, q*C_6*H_4Cl), 133.4 (1C, q*C_6*H_4Cl), 130.3 (2C, *C_6*H_4Cl), 129.7 (2C, *C_6*H_4Cl), 54.1 (1C, *C*HCHO), 52.2 (1C, *C*H_3), 34.5 (1C, *C*H_2).

Hydroformylation of 2-(4-chlorobenzylidene)malonate (**7**)

The following experiments were performed only in single determination. 2-(4-chlorobenzylidene)malonate (**7**, 0.51 g, 2.0 mmol, 0.17 M) and "[HRh(PPh_3)$_4$]" (2.5 mg, according to ICP-OES:[8] 0.11 mol% Rh) were weighed in the glass inlet which was subsequently inserted into the reactor. The autoclave was then sealed, purged with argon and toluene (12 mL) was added. The autoclave was inserted in the multi reactor system, stirred at 1000 rpm and purged with nitrogen (3x20-30 bar) and syngas (CO/H_2 (1:1), 3x20-30 bar). Then it was pressurized with 100 bar CO/H_2 (1:1) and heated to 80 °C for 15 h. Afterwards, the reactor was depressurized and purged with nitrogen (3x20-30 bar) again. From a sample of the reaction solution, the solvent was evaporated, the residue was dissolved in $CDCl_3$ and analyzed by ^{1}H NMR spectroscopy. A conversion of 96% was obtained, but only < 2% of an aldehyde signal was observed. According to NMR data from dimethyl 2-benzylmalonate of Klahn *et al*.[9], probably 94% dimethyl 2-(4-chlorobenzyl)malonate (**9**) was formed.
In the glovebox (M. Braun, argon), 2-(4-chlorobenzylidene)malonate (**7**, 0.61 g, 2.4 mmol, 0.20 M) and "[HRh(PPh_3)$_4$]" (2.7 mg, according to ICP-OES:[8] 0.096 mol% Rh) were dissolved in toluene (12 mL). 3 mL of the solution was filled in each glass inlet in the reactor. The hydroformylation was performed with 80 bar CO/H_2 at 80 °C for 15 h. The ratio of CO to H_2 was varied from (6:1 to 1:3). From a sample of the reaction solution, the solvent was evaporated and analyzed by ^{1}H NMR spectroscopy. The result is shown in Table S3.

Table S3: Hydroformylation of 2-(4-chlorobenzylidene)malonate (**7**) with 0.20 M substrate concentration. As result, the conversion and the amount of aldehyde signal is shown.

**CO/H_2 ratio**	**Pressure**	**Conversion [%]**	**Aldehyde [%]**
6:1	67 bar CO, 13 bar H_2	62	< 4
3:1	53 bar CO, 27 bar H_2	66	< 4
1:1	40 bar CO, 40 bar H_2	61	< 2
1:3	27 bar CO, 53 bar H_2	69	< 5

Ligand screening

Methyl 4-chlorocinnamate (**1a**, 0.98 g, 5.0 mmol, 1.0 M) was weight in the glass inlet which was inserted into the autoclave afterwards. The autoclave was sealed, vacuumed and purged with argon (3x). Under argon atmosphere, dry toluene (3 mL) and a solution of [Rh(acac)(CO)$_2$] (2.6 mg, 10 µmol, 0.20 mol%), tris[3,5-bis(trifluoromethyl)phenyl]phosphine (33.7 mg, 50.3 µmol, 1.01 mol%) in toluene (2 mL) was added. The reactor was inserted in the multi reactor system, stirred (1000 rpm) and purged with nitrogen (3x20-30 bar) and hydrogen (3x20-30 bar) or syngas (CO/H_2 (1:1), 3x20-30 bar). Then it was pressurized with

80 bar CO/H_2 (1:1) and heated to 80 °C. After 15 h the autoclave was depressurized and purged with nitrogen (3x20-30 bar) again. From a sample of the reaction solution, the solvent was evaporated and 40 ± 3% of β-aldehyde **2a**, 14 ± 2% of α-product **3a,** 17 ± 4% of by-product **4a**, < 1% of by-product **5** and 1% of by-product **6a** were observed by ^{1}H-NMR spectroscopy. A conversion of 72 ± 8%, a regioselectivity of 74 ± 1% and a selectivity of 55 ± 3% selectivity was determined.
With other ligands, the hydroformylation was performed with methyl 4-chlorocinnamate (**1a**, 0.59 g, 3.0 mmol, 0.60 M), [Rh(acac)$(CO)_2$] (0.8 mg, 3 µmol, 0.1 mol%) and $P(C_6H_4R')_3$ (0.5 mol%, 4-R' = H, CH_3, OCH_3, F, CF_3) analogous to the hydroformylation with tris[3,5-bis(trifluoromethyl)phenyl]phosphine. The hydroformylation with 4-R' = CH_3 and OCH_3 were performed only in single determination. The results are shown in Table S4. The amounts of formed products in percentage are present in Table S5.

Table S4: Hydroformylation of methyl 4-chlorocinnamate (**1a**) with 0.6 M substrate concentration, 0,1 mol% [Rh(acac)$(CO)_2$] and 0.5 mol% P-ligand $P(C_6H_4R')_3$ (4-R' = H, CH_3, OCH_3, F, CF_3). As result, the conversion, regioselectivity and selectivity is shown.

4-R'	Conversion [%]	Regioselectivity [%]	Selectivity [%]
OCH_3	77	37	24
CH_3	> 99	40	22
F	> 99 ± 0	46 ± 2	26 ± 2
CF_3	98 ± 0	60 ± 1	42 ± 1

Table S5: Hydroformylation of methyl 4-chlorocinnamate (**1a**) with 0.6 M substrate concentration, 0,1 mol% [Rh(acac)$(CO)_2$] and 0.5 mol% P-ligand $P(C_6H_4R')_3$ (4-R' = H, CH_3, OCH_3, F, CF_3). As result, the percentage of all detected products are shown. α-aldehyde **3a** includes the tautomeric α-enol.

4-R'	2a [%]	3a [%]	4a [%]	5 [%]	6a [%]
OCH_3	18	32	26	< 1	< 1
CH_3	22	34	43	< 1	< 1
F	26 ± 2	31 ± 1	42 ± 3	< 1	< 1
CF_3	41 ± 1	28 ± 1	27 ± 2	1	1

2.4 Isolation of product and scale-up

Purification by bisulfite adduct formation
In single determination, methyl 4-chlorocinnamate (**1a**, 2.05 g, 10.4 mmol, 0.61 M) and "[HRh$(PPh_3)_4$]" (5.9 mg, according to ICP-OES:[8] 0.048 mol% Rh) were weighed in the glass inlet which was subsequently inserted into the reactor. The autoclave was then purged with argon. Under an argon atmosphere, toluene (17 mL) was added. The autoclave was inserted in the multi reactor system, stirred at 1000 rpm and purged with nitrogen (3x20-30 bar) and syngas (CO/H_2 (1:1), 3x20-30 bar). Then it was pressurized with 80 bar CO/H_2 (1:1) and heated to 80 °C for 15 h. Afterwards, the reactor was depressurized and purged with nitrogen (3x20-30 bar) again. The solution was filtered over celite and the solvent was evaporated. A crude product (1.59 g) was obtained and analyzed by ^{1}H-NMR spectroscopy. 56%

β-aldehyde **2a**, 6% α-product **3a**, 24% product **4a** and 14% substrate **1a** were observed resulting in a conversion of 85%, a regioselectivity of 91% and a selectivity of 66%.
For the bisulfite adduct formation, 0.40 g crude product was dissolved in ethanol (3.5 mL) and a solution of sodium bisulfite (234 mg, 1.23 mmol) in water (0.35 mL) was added. The reaction mixture was stirred for 18 h at 60 °C and incubated for 1.5 h at -20 °C. The suspension was filtered, and the resulting brownish solid was washed with *n*-hexane. One half of the solid was stirred in EtOAc, isolated, dried. Afterwards, the solid (7 mg) was incubated in a two-phase system of dH_2O (29 µL) and toluene (29 µL) for 1.5 h at room temperature in an Eppendorf shaker at 800 rpm. Subsequently, centrifugation at 20.000 g was performed to improve phase separation. The organic phase was then washed with a saturated NaCl solution, centrifuged again, and the solvent was removed. The isolated product (2 mg, 9 µmol, 1% yield) was analyzed by ^{1}H-NMR spectroscopy in $CDCl_3$ and yielded a mixture of 74% β-aldehyde **2a** and 26% α-product **3a**.

Purification by column chromatography
In single determination, methyl 4-chlorocinnamate (**1a**, 0.98 g, 5.0 mmol, 1.0 M) and "[$HRh(PPh_3)_4$]" (5.7 mg, according to ICP-OES:[8] 0.097 mol% Rh) were weighed in the glass inlet which was subsequently inserted into the reactor. The autoclave was purged with argon. Under an argon atmosphere, toluene (5 mL) was added. The autoclave was stirred at 1000 rpm and purged with nitrogen (3x20-30 bar) and syngas (CO/H_2 (1:1), 3x20-30 bar). Then it was pressurized with 70 bar CO/H_2 (1:1) and heated to 100 °C for 15 h. Afterwards, the reactor was depressurized and purged with nitrogen (3x20-30 bar). The solution was filtered over celite and the solvent was evaporated. A yellow oil was obtained as crude product (0.79 g) and analyzed by ^{1}H-NMR spectroscopy. 52% β-aldehyde **2a**, 6% α-product **3a**, 29% product **4a**, 12% substrate **1a** and < 1% by-product **5** and **6a** were observed resulting in a conversion of 88%, a regioselectivity of 90% and a selectivity of 59%.
The crude product was purified by column chromatography (1 CV 5% EtOAc, 15 CV 5-40% EtOAc, 8 CV 40% EtOAc, 40 mL/min Sfaer Silica 10 g HC D column) and β-aldehyde **2a** was obtained (0.36 g, 1.6 mmol, 32% yield) with a purity of 93% (7 % α-product **3a**).

^{1}H-HMR (500 MHz, $CDCl_3$) of β-aldehyde **2a**: *δ* [ppm] = 9.68 (s, 1H, C*H*O), 7.36 (m, 2H, C_6H_4Cl), 7.14 (m, 2H, C_6H_4Cl), 4.13 (dd, J = 7.9, 6.4 Hz, 1H, C*H*CHO), 3.67 (s, 3H, CH_3), 3.15 (dd, J = 16.9, 8.0 Hz, 1 H, C*H*), 2.60 (dd, J = 16.9, 6.4 Hz, 1 H, C*H*).
Accurate mass (ESI): $[C_{11}H_{11}ClO_3 + Na]^+$ *m/z* (calcd): 249.02889, *m/z* (found): 249.029.

Scale-up and isolation of methyl 3-(4-chlorophenyl)-4-oxobutanoate (**2a**)
In single determination, methyl 4-chlorocinnamate (**1a**, 2.40 g, 12.2 mmol, 0.61 M) and [$Rh(acac)(CO)_2$] (3.1 mg, 12 µmol, 0.098 mol%) were weighed in the glass inlet which was inserted into the reactor. The autoclave was purged with argon and under an argon atmosphere, toluene (17 mL) was added. The autoclave was stirred at 1000 rpm and purged with nitrogen (3x20-30 bar) and syngas (CO/H_2 (1:1), 3x20-30 bar). Then it was pressurized with 80 bar CO/H_2 (1:1) and heated to 80 °C for 15 h. Afterwards, the reactor was depressurized and purged with nitrogen (3x20-30 bar) again. The solution was filtered over celite and the solvent was evaporated. A crude product (2.56 g) was obtained with 83% conversion, 91% regioselectivity and 77% selectivity. Purification by column chromatography (1 CV 5% EtOAc, 20 CV 5-40% EtOAc, 3 CV 40% EtOAc, 80 mL/min Sfaer Silica 25 g HC D column) yielded methyl 3-(4-chlorophenyl)-4-oxobutanoate (**2a**, 1.13 g, 4.99 mmol, 41% yield) with a purity of 92% (8% α-aldehyde **3a**).

^{1}H-NMR (500 MHz, $CDCl_3$): *δ* [ppm] = 9.68 (s, 1H, C*H*O), 7.36 (m, 2H, C_6H_4Cl), 7.14 (m, 2H, C_6H_4Cl), 4.13 (dd, J = 7.9, 6.4 Hz, 1H, C*H*CHO), 3.67 (s, 3H, CH_3), 3.15 (dd, J = 16.9, 8.0 Hz, 1 H, C*H*), 2.60 (dd, J = 16.9, 6.4 Hz, 1 H, C*H*).

2.5 Enzyme Screening

Transaminase gene expression in 96-well microtiter plates

The inhouse transaminase library contained various (*R*)- and (*S*)-selective amine transaminase genes, most of them are described in previous publications.[10–24] The genes are inserted in pET28b, pET22b or pGASTON plasmids, which are inducible with IPTG or rhamnose and contained an ampicillin or kanamycin selection marker. The library was stored as glycerol stocks of *E. coli* BL21 DE3 transformants in 96-well microtiter plates at -80°C, grouped by antibiotic and inducer.

To prepare an overnight culture, a 96-well plate containing 200 µL LB medium per well and the appropriate antibiotic for selection was inoculated from the glycerol stock plate using a 96-tip replicator. This preculture was incubated for approx. 18 h at 30°C and 600 rpm. For expression, a 96-deep well block was prepared with 1 mL of TB medium and the appropriate antibiotic per well. Inoculation was done by adding 100 µL of the overnight culture. After a four-hour incubation at 30°C and 600 rpm, either 0.2% Rhamnose (w/v) or 0.1 mM IPTG was added for induction. The expression was carried out for about 18 h at 20°C and 600 rpm. The cells were then harvested by centrifugation at 4369 g and 4°C for 40 min. If not used immediately, the resulting pellets were stored after sealing with a semi-permeable membrane (Thermo Fisher Scientific) at -20°C.

Purification and desalting in 96-well microtiter plates

To lyse the cell pellets, 300 µL of lysis buffer was added to each well, which was freshly prepared by adding 0.1 mM PLP, 1 mg/mL lysozyme and 1µg/mL DNaseI in 50 mM HEPES buffer, pH 8. The plate was incubated for 1 h at 30°C and 800 rpm and centrifuged for 40 min at 4°C and 4369 g. 200 µL of the resulting supernatant was transferred to a fresh microtiter plate for further use or stored at 4°C until further processing of the lysates.

For purification, 96-well HIS-Select filter plates (Sigma-Aldrich / Merck) were used, and the protocol of the manufacturer was followed: A HIS-Select Filter Plate was placed in a collection plate and equilibrated by pipetting 600 µL of equilibration buffer (50 mM HEPES, pH 7.5, 300 mM NaCl, 15 mM imidazole) per well and centrifuging at 4369 g for 2 min at room temperature. The emptied collection plate was placed back under the filter plate, before loading the cell extracts (270 µL per well). After centrifugation for 2 min, the flowthrough was reloaded onto the filter plate to ensure complete binding of the proteins, and after the centrifugation, the filter plate was washed two times by application of 600 µL of equilibration buffer and subsequent centrifugation. Using a new collection plate, the proteins were eluted two times by applying 300 µL of elution buffer (50 mM HEPES, pH 7.5, 300 mM NaCl, 300 mM imidazole) followed by centrifugation for 2 min.

Desalting was conducted by employing the 96-well Zeba Spin Desalting plates (Thermo Scientific) by following the instructions of the manufacturer. For the first use, the plates were centrifuged at 1000 g for 2 min to remove the storage buffer. For equilibration, the plates were loaded three times with 250 µL of equilibration buffer (50 mM HEPES pH 7.5, 150 mM NaCl) followed by centrifugation at 1000 g for 2 min, and then loaded with 100 µL of the protein eluate from the HIS-Select filter plates per well. After centrifugation at 1000 g for 2 min, the desalted proteins were in the flowthrough and were collected. The plate was equilibrated again and used for desalting of the remaining protein eluate from the HIS-Select filter plates. The desalted proteins were then stored at 8 °C until further use.

Alanine dehydrogenase assay

For the enzyme screening we adopted the alanine dehydrogenase assay published earlier[12] To increase the signal to noise ratio, purified and desalted ATA solutions were employed rather than cell extract (which would also be possible if the enzyme activity is sufficiently high). Furthermore, the assay reaction was conducted as a discontinuous (end point) assay and was divided into two stages.

First stage: transaminase reaction. The purified ATA is incubated with the substrate mix. In case of activity, the amino group of the amine substrate Baclofen or Baclofen methylester is transferred onto pyruvate to yield L-alanine. When (*R*)-selective ATA are assayed, an alanine racemase is included in the substrate mix so that the produced D-alanine is isomerized). Depending on the amount of transaminase obtained in the expression and its activity, this first step was conducted for 1 h or overnight (16 h).

Second stage: detection reaction. The detection mix is then added, and L-alanine dehydrogenase converts the formed L-alanine to pyruvate and NADH, which then transfers its electrons via methoxy-PMS to the tetrazolium salt XTT to yield the colored formazane dye. This two-step procedure ensured that low ATA activities can be detected: A significant amount of L-alanine is accumulated in the first incubation step, which is then converted into the colored product. A further advantage is that a lower amount of alanine racemase is sufficient to perform the racemization step. The assay can also be conducted in the concurrent way (all enzymes and reagents added at the beginning). However, there is a significant NADH background oxidation (especially when cell extract is used as the ATA enzyme source). Therefore, a signal is only visible if the ATA activity (and the formation of NADH by alanine dehydrogenase) exceeds the background NADH oxidation.

For the assay, the following solutions are prepared in 50 mM HEPES buffer, pH 8.

Detection solution: For assaying one 96-well plate, approx. 7.2 mL detection solution are necessary. The following reagents are added to 10 mL HEPES buffer: 13,3 mg NAD^+, 50 µL of a 1 mM methoxy-PMS stock solution (3.4 mg in 10 mL A. dest, can be stored at -20 °C), 50 µL XTT stock solution (40 mg in 1 mL DMSO, stored at -20 °C), 110 µL alanine dehydrogenase stock solution (5 mg alanine dehydrogenase recombinant, Merck KGaA, Darmstadt) in 0,5 mL HEPES buffer and 0.5 mL glycerol, stored at -20 °C).

The **Substrate solution** is composed of 5 mM amine donor (either *rac*-Baclofen, *rac*-Baclofen methylester, or as a control substrate: *rac*-1-phenylethaneamine or rac-hexane-2-amine), and 2 mM sodium pyruvate, dissolved in HEPES buffer containing 4,5 %(v/v) DMSO.

The assay procedure is as follows:

Step 1: Transaminase reaction:

Per well 75 µL of substrate solution is mixed with 50 µL of the purified desalted ATA enzyme solution in an appropriate dilution. For the assays of the (*R*)-selective ATA, 0.08 U/well alanine racemase (from *Bacillus stearothermophilus,* Merck KGaA, Darmstadt), was added per well. The plate is then incubated at 30 °C, 400 rpm for 1 h.

Step 2: Alanine dehydrogenase reaction:

Per well of the enzyme reaction, 75 µL of detection mix is added to each well and the plate is incubated again for 1 h at 30 °C at 400 rpm, before measuring the absorbance at 470 nm. As a blank (negative control), the purified enzyme solution was replaced by HEPES buffer. As positive controls, *rac*-1-phenylethaneamine was employed as amino donor, as most ATA convert this model substrate with high efficiency.

The reactions were performed in duplicates. The mean values were calculated and reduced by the mean value of a blank reaction. A calibration curve was done in the following way: per well, 125 µL of solutions with different L-alanine concentrations in HEPES buffer was mixed with 75 µL of the detection mix to give L-alanine end concentrations between 5 µM – 300 µM. The plate is incubated again for 1 h at 30 °C at 400 rpm, before measuring the absorbance at 470 nm. The concentrations of L-alanine formed in the assay reactions were calculated with the formula obtained after linear regression of the signals from the alanine standard curve. Note that for (*R*)-selective ATA, the observed signal in the assay is only half of that with an (*S*)-selective ATA because the L-alanine concentration generated upon a certain conversion is 50 % smaller due to the racemization.

<u>Acetophenone assay</u>

The acetophenone assay was adapted from Schätzle et al.[25] In a UV-transparent 96-well microtiter plate, 195 µL of the master mix was added (2.5 mM (*S*)- or (*R*)-1-phenylethaneamine, 2.5 mM β-aldehyde **2a**, 0.1 mM PLP in 50 mM HEPES buffer, pH 8)

was added. The final DMSO concentration was 1% (v/v). The plate was preincubated at 30 °C for 5 min, before addition of 5 µL of the purified and desalted enzyme solution at a reasonable dilution per well. Absorbance increase at 245 nm correlating to acetophenone level increase was recorded for 20 min (at 30 s intervals) at 30 °C. The following controls were used: replacement of the enzyme solution by HEPES buffer (blank), and a sample not containing aldehyde **2a**. One unit (U) activity was defined as the formation of 1 µmol formed acetophenone per minute. All measurements were performed in triplicates.

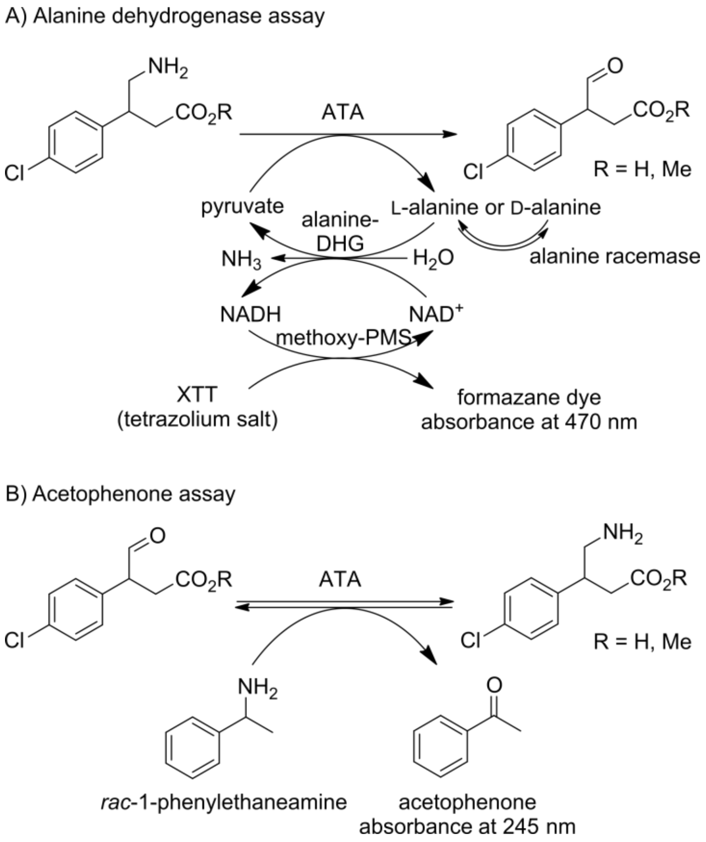

Scheme S1: Photometric enzyme screening assays used to identify active amine transaminases. **A)** In the alanine dehydrogenase (alanine DHG) assay, NADH is formed in case of ATA activity by a coupled enzyme cascade, which then acts as a reductant via the redox mediator 1-methoxy-5-methylphenaziniummethyl-sulfat (methoxy-PMS) and converts the colorless 2,3-bis-(2-methoxy-4-nitro-5-sulfophenyl)-2H-tetrazolium-5-carboxanilid XTT into a highly absorbing formazan dye. **B)** The co-product is detected directly in the acetophenone assay.

2.6 Preparation of biocatalysts

Transformation

50 µl aliquots of chemocompetent *E.coli* BL21(DE3) cells were thawed on ice for approximately 10 min. To the thawed cell suspension on ice 1 µl of the plasmid pET-22b(+) carrying the gene for ATA 117 from *Arthrobacter sp. KNK168* (for amino acid sequence, see below) with a C-terminal 6xHis-tag was added. The cells were incubated for 30 min on ice. After that, the heat shock was performed by placing the cell aliquot into a prewarmed heating block at 42 °C for 30s. The cells were placed back onto the ice immediately after the heat shock and incubated for another 5 min. 950 µl of sterile LB medium were added to the cells. This suspension was incubated at 37 °C and 750 rpm for 1 hour. Variable volumes were plated onto selective LB-agar plates (ampicillin selection). The plates were incubated at 37 °C overnight.

ATA 117wt amino acid sequence:
MAFSADTSEIVYTHDTGLDYITYSDYELDPANPLAGGAAWIEGAFVPPSEARISIFDQGYLHS
DVTYTVFHVWNGNAFRLDDHIERLFSNAESMRIIPPLTQDEVKEIALELVAKTELREAFVSVS
ITRGYSSTPGERDITKHRPQVYMYAVPYQWIVPFDRIRDGVHAMVAQSVRRTPRSSIDPQV
KNFQWGDLIRAVQETHDRGFEAPLLLDGDGLLAEGSGFNVVVIKDGVVRSPGRAALPGITR
KTVLEIAESLGHEAILADITLAELLDADEVLGCTTAGGVWPFVSVDGNPISDGVPGPVTQSIIR
RYWELNVESSSLLTPVQY

Overnight cultures and cryostocks

Overnight cultures were prepared in 5 ml of sterile LB medium and supplemented with ampicillin to a final concentration of 0.1 mg/ml or kanamycin to a final concentration 0.05 mg/ml. The cultures were inoculated from either single colonies or cryostocks and grown overnight at 37 °C and 170 rpm.

Cryostocks were prepared by adding 800 µl of an overnight culture to 200 µl of sterile glycerol (resulting in 20% v/v glycerol stocks) and frozen at - 80 °C.

Protein overexpression

Expression cultures were grown in 500 ml terrific broth (TB) medium in 2 l cultivation flasks. 1 ml of 50 mg/ml ampicillin stock solution (0.1 mg/ml final concentration) or 500 µl of 50 mg/ml kanamycin stock solution (0.05 mg/ml final concentration) was added. Further, 500 µl of a sterilized 1:10 dilution of antifoam B (Sigma) were added to prevent foaming. The flasks were inoculated to a starting OD_{600} of approximately 0.05 from overnight cultures. The cultures were incubated at 37 °C for approximately 4 hours under constant shaking at 200 rpm, until they reached an OD_{600} of approximately 0.6-0.8. Then, the cultures were induced with 0.5 mM IPTG (250 µl of sterile 1 M IPTG stock in ddH_2O per flask) and left shaking at 200 rpm at 20°C overnight for the overexpression step.

After cultivation the cells were harvested via centrifugation at 4000xg for 10 min at 4 °C. The pellets were resuspended in 50 ml of 50 mM HEPES buffer (pH 7.5) with 0.1 mM PLP and centrifuged again at 4000xg for 10 min at 4 °C, the liquid was discarded. After resuspension in 5-10 ml of 50 mM HEPES buffer (pH 7.5) with 0.1 mM PLP, the pellet suspensions were unified and lyophilized.

TB medium preparation: The following components were mixed together in the cultivation flasks: 450 ml ddH_2O, 6 g tryptone, 12 g yeast extract and 2.5 g glycerol and the resulting solution was sterilized by autoclave. After the cooling to RT, 50 ml of autoclaved 1 M potassium phosphate buffer (125.4 g K_2HPO_4, 23.1 g KH_2PO_4 in 1 l of ddH_2O, pH adjusted to 7.2) and the appropriate antibiotic were added before usage.

Transaminase activity assay

The measurements were performed with a Specord 50 plus spectrophotometer from Analytik Jena (Jena, Germany) at a wavelength of 245 nm (acetophenone extinction coefficient of 11.852 $(mM\cdot cm)^{-1}$). 50 mM sodium phosphate buffer with 0.25 % (v/v) DMSO was adjusted to pH 8 with saturated NaOH solution and conc. HCl and used for all further solutions. 250 µl of the buffer solution, 250 µl of a 10 mM (*R*)- (for ATA 117wt) or (*S*)-1-phenylethylamine solution in buffer and 250 µl of a 10 mM sodium pyruvate solution in buffer were mixed in the measurement cuvettes. The enzyme samples were prepared by dissolving 1 mg of dry weight whole cells in 1800 µl of buffer and adding 200 µl of a 10 mM pyridoxal phosphate solution in buffer. 250 µl of this enzyme mix were then added to the previously prepared solution mix, briefly vortexed and measured immediately. All experiments were measured against a reference solution by replacing the enzyme mix with 50 µl of the 10 mM pyridoxal phosphate and 200 µl of buffer. Specific enzyme activity was calculated through the slope of acetophenone extinction over the course of 1 min. One unit was defined as the conversion of 1 µmol of (*S*)-1-phenylethylamine to acetophenone per minute.

2.7 Biocatalytic reactions

Reactions in 1 ml format

For 1 ml reactions, a reaction stock solution of 10 ml 50 mM HEPES buffer pH 8 with 250 mM IPA and 2.5 mM PLP. The pH was adjusted to the desired value (between 7 and 10 depending on the experiment) with conc. HCl and saturated NaOH solutions.

The appropriate amount of dry-weight whole cells (either 80 U/ml or 20 U/ml) was weighed into 2 ml microcentrifuge tubes. To those cells, 1 ml of the premixed stock solution was added and the tubes were thoroughly vortexed. The pH was checked and readjusted back to the desired value, if necessary. The reactions were started by the addition of 100 mM of the hydroformulation substrates **10** (17.5 µl/ml, density approx. 1.1 g/ml) or **2a** (20.6 µl/ml, density approx. 1.1 g/ml) and shaken at 30 °C for 24 hours and 900 rpm. For inhibition tests, 50 mM of the hydration adduct (**4**) was added to the reactions with **10**. 2 separate 100 µl samples were drawn for quantification of acetophenone (**12**) and lactone by-product (**13**) formation (one sample) and product lactam (**14**) formation (one sample). The samples were processed as described in the Gas chromatography section.

With the fed-batch approaches, two parallel reactions were started with the same reaction conditions. After 24 hours from one of the reactions, a 50 µl sample was drawn for quantification of **12a** and **13a**, the rest of the reaction was completely extracted for quantification of **14a**. After quantification, the second parallel reaction was refilled with **2a** according to the quantification of **12a**, **13a** and **14a** (sum of formed product and byproducts, 1 mM substrate consumed per 1 mM product or 1 mM byproduct) and additional dry-weight whole cells (20 U/ml; if necessary). IPA was refilled according to the quantification of **14a** to reach the starting concentration of 250 mM again (1 mM IPA consumed per 1 mM product). After 48 hours, the complete reaction was extracted for quantification of the total product amine amount. Sample processing is described in the Gas chromatography section.

Hydrolysis of extracted product lactams

Wholly extracted 1 ml reactions were hydrolyzed in CPME. The CPME extraction phase was divided into two 2 ml Eppendorf tubes (approx. 650 µl per tube). 600 µl of 3 M HCl in CPME was added per tube. The mixtures were shaken at 900 rpm at 100 °C for approximately 6 h. Then, the residual CPME was evaporated. The hydrolysis product was analyzed via chiral HPLC.

2.8 Analysis of biocatalytic reaction products

Synthesis of baclofen methyl ester (11a) for gas chromatography calibration

Racemic baclofen (200 mg, 0.936 mmol) was dissolved in 40 ml HPLC-grade methanol. 210 µl of thionyl chloride were added. The reaction was stirred for 4.5 hours at 35 °C and 200 rpm under reflux. Residual methanol was evaporated to yield the hydrochloride of the baclofen methyl ester (245 mg, 0.927 mmol, 93 % conversion – ^{13}C-NMR). The calibration of the gas chromatography was performed through the preparation of differently concentrated baclofen methyl ester hydrochloride solutions and their basic extraction according to the procedure described in the "Gas chromatography" part.

^{1}H-NMR (400 MHz, D_2O): *δ* [ppm] = 7.39-7.25 (m, 4H, C_6H_4Cl), 3.50 (s, 3H, OCH_3), 3.43-3.38 (quin, J=5.45 Hz, 1H, CHR_2), 3.30-3.19 (ddd, 2H, $CH_2NH_3^+$), 2.85-2.68 (ddd, 2H, RCH_2R).

^{13}C-HMR (100.6 MHz, D_2O): *δ* [ppm] = 173.87 (s, 1C, RCOOR), 136.86 (s, 1C, ArylC-CHR_2), 133.42 (s, 1C, ArylC-Cl), 128.42-128.27 (m, 4C, Aryl), 52.31 (s, 1C, $ROCH_3$), 43.53 (s, 1C, $RCH_2NH_3^+$), 39.43 (s, 1C, $RCHR_2$), 38.09 (s, 1C, RCH_2R).

Gas chromatography

All reactions were monitored via gas chromatography. For measurement preparations, 50 µl or 100 µl samples were drawn from the reactions. Samples for acetophenone quantification were left pure. For amine quantification, 20 µl (200 µl for wholly extracted reactions) of saturated sodium hydroxide solution were added to facilitate amine deprotonation. Two samples were drawn for one measurement.

70 µl (for 50 µl samples) or 140 µl (for 100 µl samples) of CPME were added per sample. With this, an extraction was performed by vortexing the samples for 1 minute at max. speed. For wholly extracted reactions, the same proportions would apply (1330 µl of CPME for 950 µl of reaction).

After phase separation, 50 µl of the CPME-phase were drawn for GC-analysis. This sample aliquot was added to 50 µl of pure CPME and 20 µl of 25 mM n-decane solution in CPME as an internal standard, thus yielding a 120 µl sample for GC-measurements.

The measurements were performed on a Shimadzu Nexis 2030 gas chromatograph equipped with the AOC 20i Plus autoinjection module and a flame ionization detector (FID). A SH-5 column (0.25 mm x 30 m x 0.25 µm, SH-5 phase) by Shimadzu was used. Hydrogen with nitrogen as a make-up gas and synthetic air were used as the carrier.

The following temperature program was used: 90 °C; 1. 90-100 °C: rate of 2 °C/min; 2. 100-130 °C: rate of 20 °C/min; 3. 130-250 °C: rate of 10 °C/min; 4. Hold 250 °C for 3 min; Split factor 10.0. The high split factor was applied for better product quantification. The injection volume was 1 µl.

The chromatograms were refined and analyzed via the LabSolutions software by Shimadzu. The amine peak areas were normalized with the internal standard peak area, then scaled for an internal standard peak area value of 1000000 sqU and then the amine concentration was calculated according to the calibration parameters.

Chiral HPLC

Chiral HPLC analysis was performed on a Shimadzu Nexera series HPLC consisting of the following modules: SCL-40, DGU-405, LC-40D, SIL-40C, CTO-40S, SPD-M40. The HPLC was equipped with a Phenomenex Chirex 3126 column (150 x 4.6 mm; 5 µm, 110 Å). The diluent was a mixture of 90% of 2 mM $CuSO_4$ solution in ddH_2O and 10% of HPLC-grade isopropanol.

Approximately 3-5 mg of the hydrolysis product were dissolved in 1 ml of diluent. The samples pH was kept over pH 3 and, if needed, adjusted with saturated NaOH and conc. HCl in order to not damage the column. The sample was measured in isocratic mode at a flow rate of 1 ml/min for 75 min to flush down any unhydrolyzed reaction product. The injection volume was 10 µl. Column temperature was kept at 30 °C. The chromatograms were refined using the LabSolutions software by Shimadzu.

2.9 NMR spectra

The ^{1}H-NMR spectroscopic data for α-aldehyde **3a** and 3-(4-chlorophenyl)propanal (**5**) are shown in our recent publication.[26]

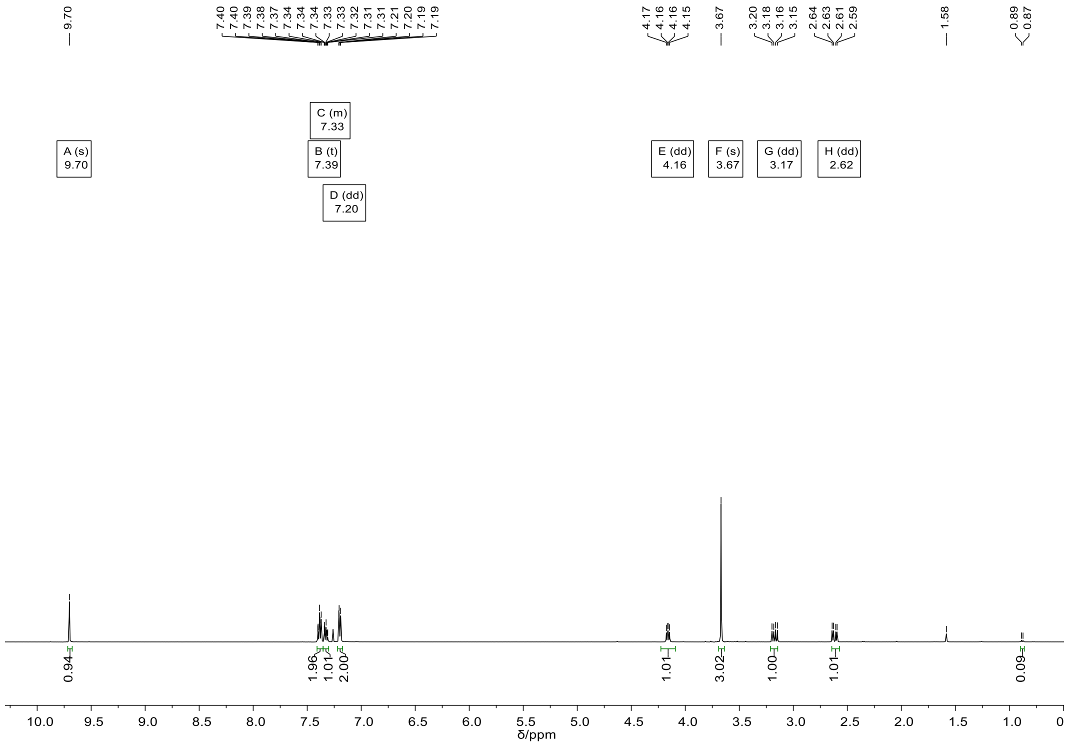

Fig. S1: ^{1}H-NMR (500 MHz) spectrum of methyl 4-oxo-3-phenylbutanoate (**10**) after column filtration in $CDCl_3$.

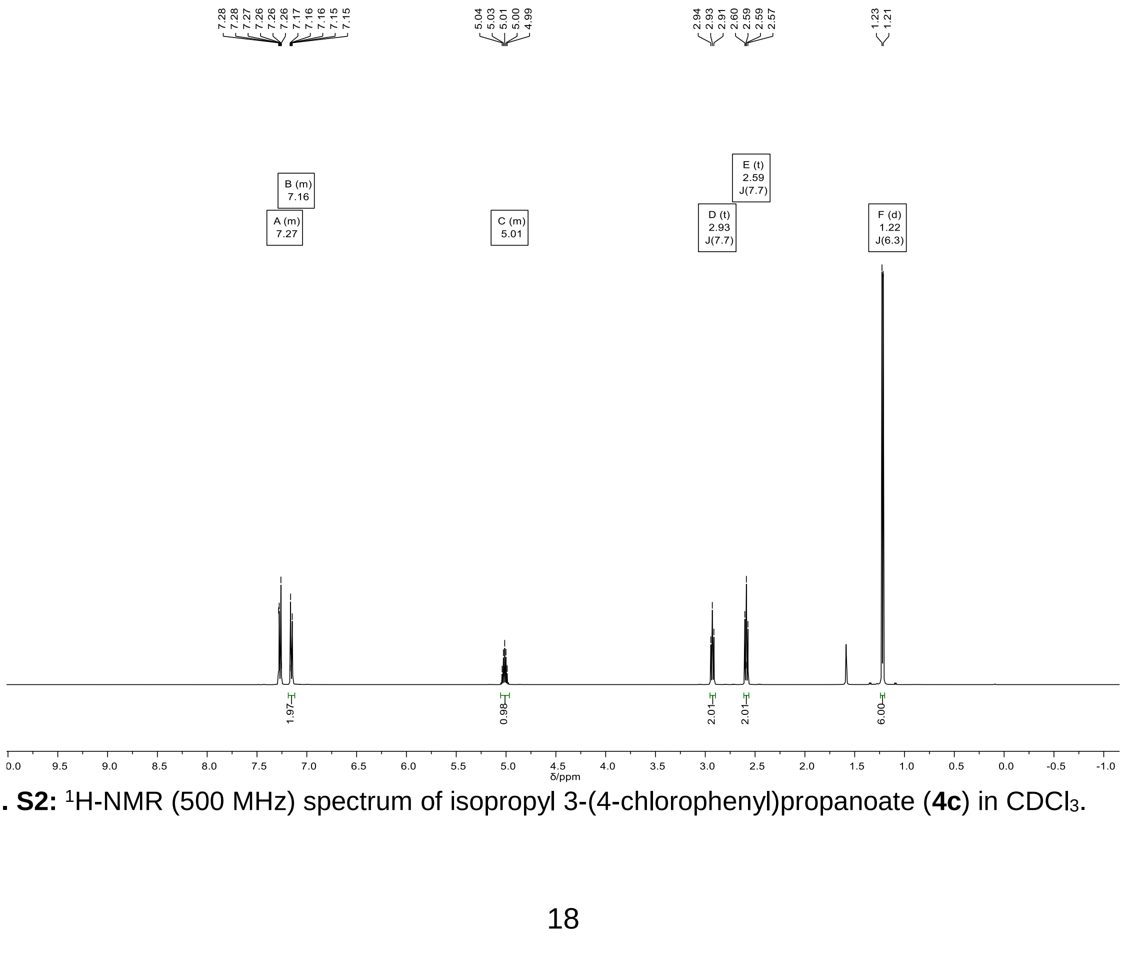

Fig. S2: ^{1}H-NMR (500 MHz) spectrum of isopropyl 3-(4-chlorophenyl)propanoate (**4c**) in $CDCl_3$.

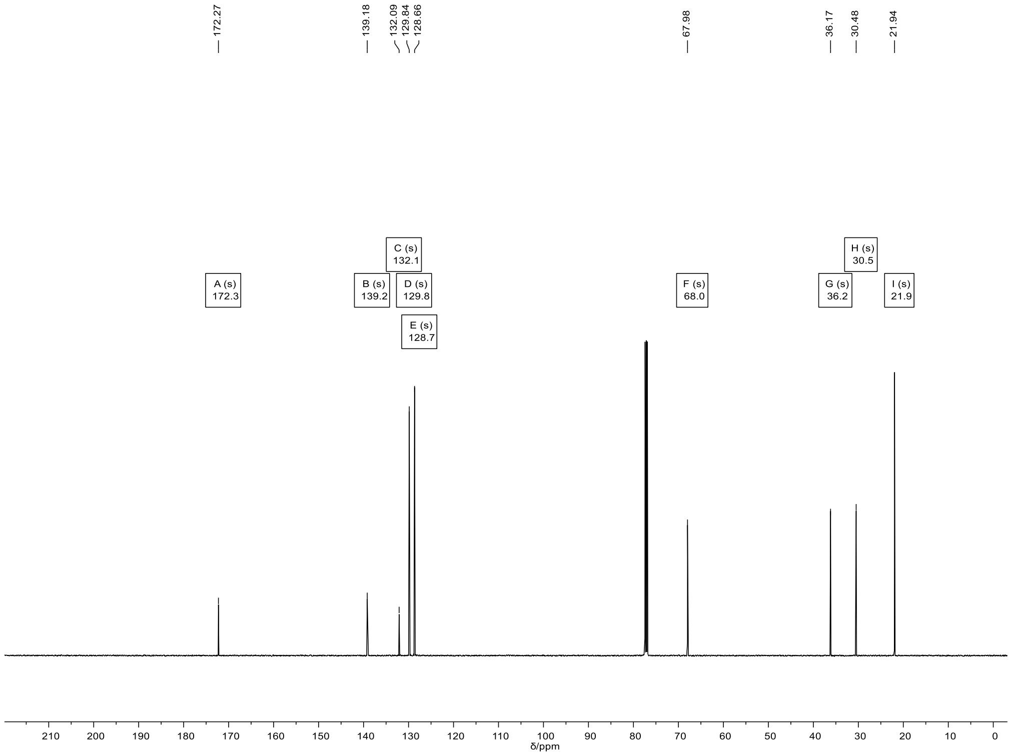

Fig. S3: $^{13}C\{^1H\}$-NMR (126 MHz) spectrum of isopropyl 3-(4-chlorophenyl)propanoate (**4c**) in $CDCl_3$.

By-product **6a**

An additional by-product (by-product **6a**) was observed with ≤ 3% related to the NMR integral in ^1H-NMR spectrum. The ^1H and $^{13}C\{^1H\}$-NMR spectra of by-product **6a** (with β-aldehyde **2a**) are shown in Fig. S and Fig. S. Both spectra indicate that the structure of by-product **6a** is closely related to methyl 3-(4-chlorophenyl)propanoate (**4a**), but with an unknown substituent replacing one proton from each CH_2-group.

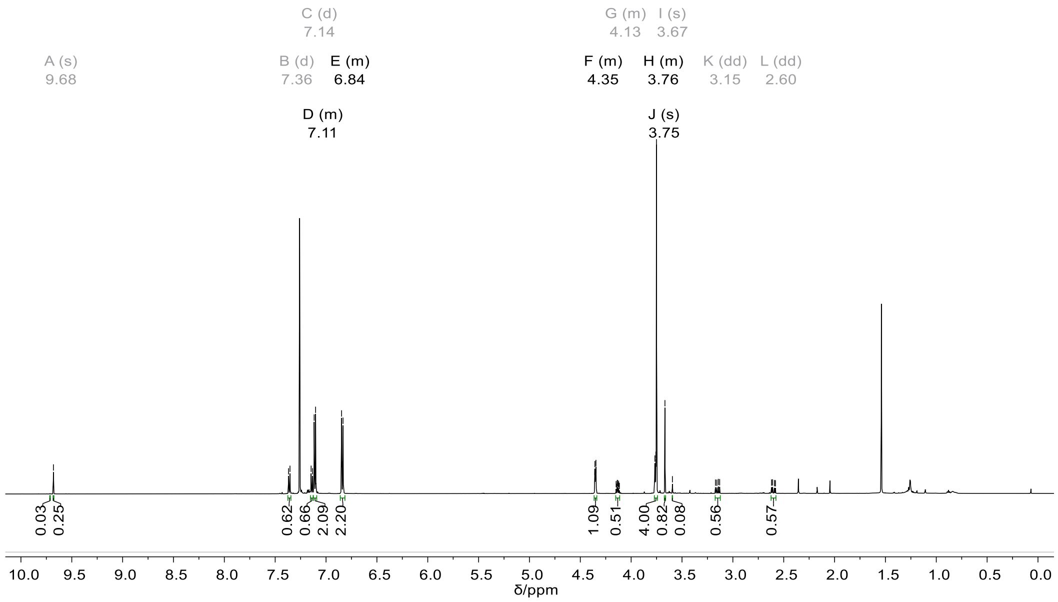

Fig. S4: ^1H-NMR (600 MHz) spectrum of by-product **6a** and β-aldehyde **2a** (RT) in $CDCl_3$. In grey, the signals from β-aldehyde **2a** are marked, while the signals from by-product **6a** are shown in black color. This figure was already reported by us recently.[26]

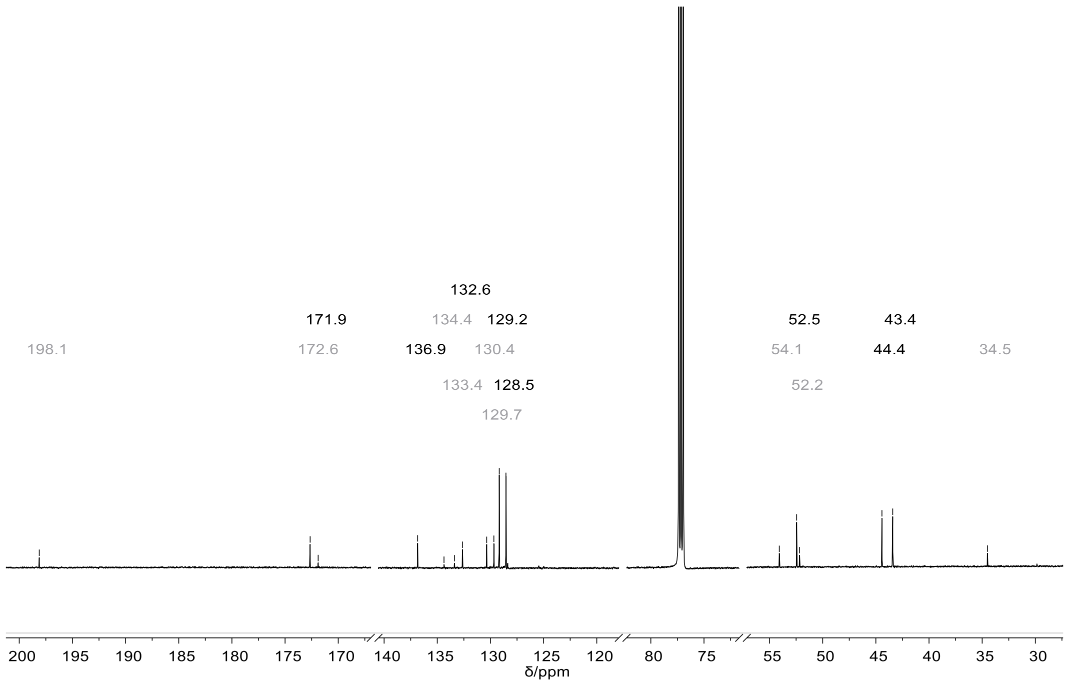

Fig. S5: $^{13}C\{^1H\}$-NMR (600 MHz) spectrum of by-product **6a** and β-aldehyde **2a** (RT) in $CDCl_3$. In grey, the signals from β-aldehyde **2a** are marked, while the signals from by-product **6a** are shown in black color. This figure was already reported by us recently.[26]

<u>By-product **13b**</u>

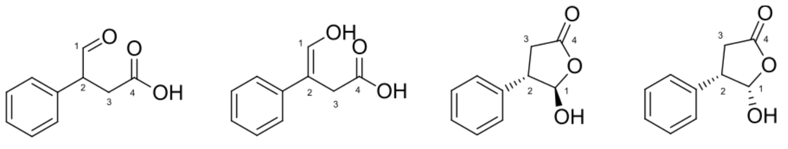

Aldehyde (25%) Enol (1%) β-Hemiacetal (60%) α-Hemiacetal (14%)

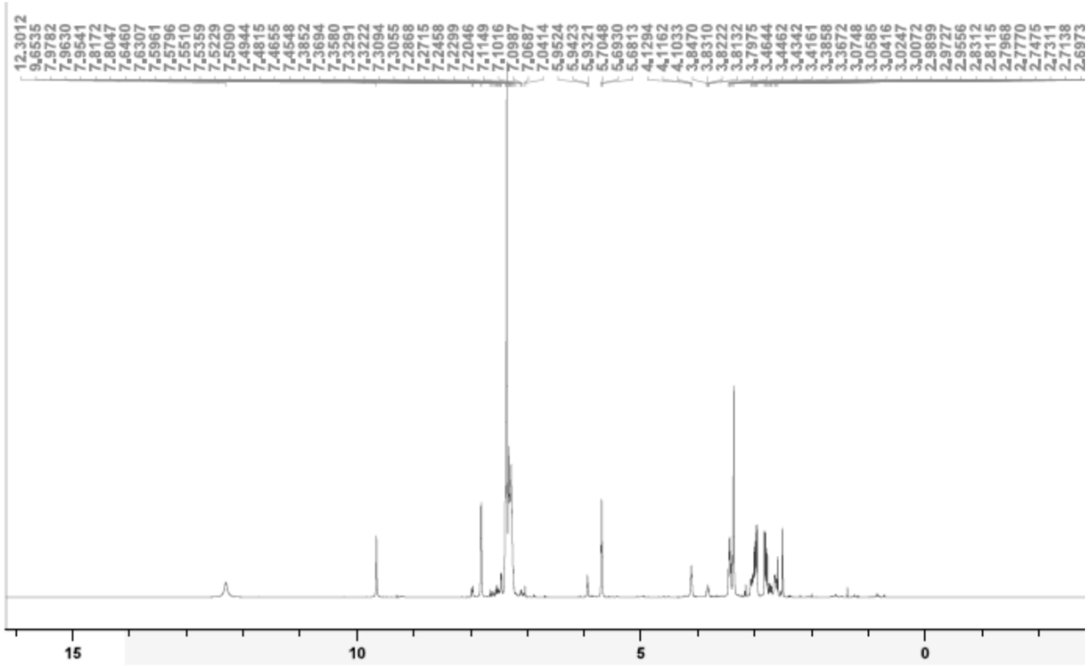

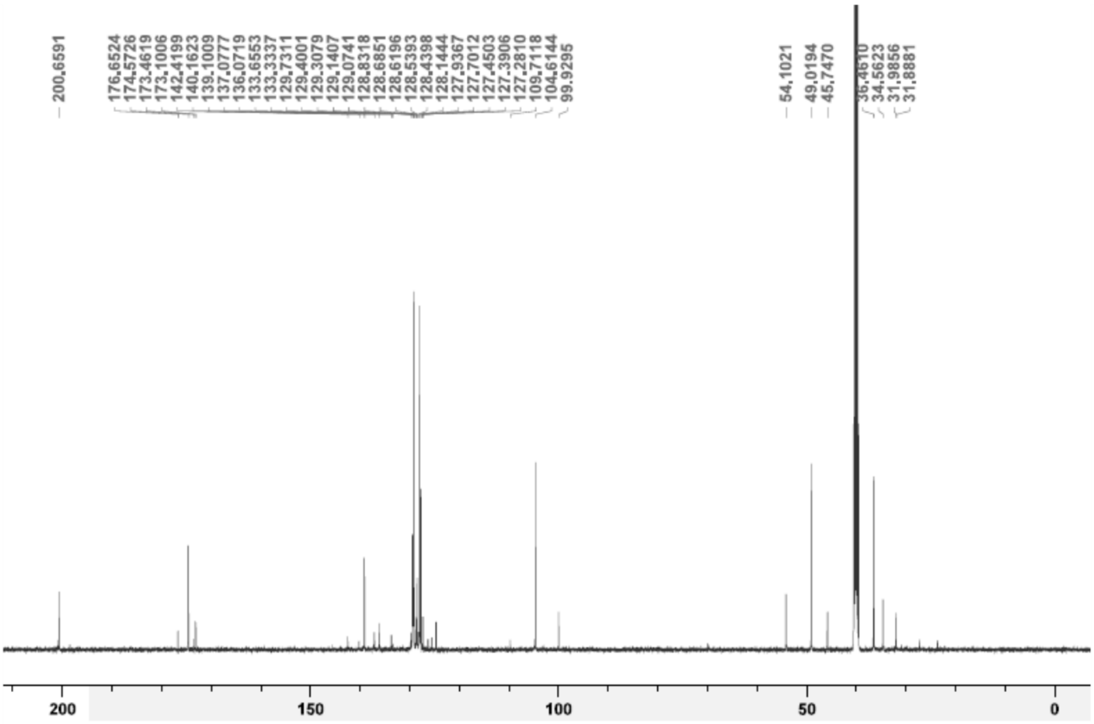

Fig. S6: Configuration of by-product **13b** and its ^{1}H-NMR (500 MHz) and ^{13}C-NMR (125 mHz) spectra. The by-product was isolated from reactions with the substrate **10**.

^{1}H-NMR (500 MHz, DMSO-d_6, 2.5ppm):
β-Hemiacetal (*threo*) (60%): *δ* [ppm] = 7.80 (d, $^{3}J_{H-1,OH}$ = 6.3 Hz, 1H, OH); 7.41-7.21 (m, Ph); 5.69 ('t', $^{3}J_{H-1,OH}$ = 6.3, $^{3}J_{H-2,H-1}$ = 6.0 Hz, 1H, C*H*OH (H-1)); 3.44 ('t'd, $^{3}J_{H-3b,H-2}$ = 9.9 Hz, $^{3}J_{H-3a,H-2}$ = 8.7 Hz, $^{3}J_{H-2,H-1}$ = 6.0 Hz, 1H, *CH*CH$_2$ (H-2)); 2.97 (dd, $^{2}J_{H-3a,H-3b}$ = 17.3 Hz, $^{3}J_{H-3a,H-2}$ = 8.7 Hz, 1H, C*H*H (H-3a)); 2.80 (dd, $^{2}J_{H-3a,H-3b}$ = 17.3 Hz, $^{3}J_{H-3b,H-2}$ = 9.9 Hz, 1H, CH*H* (H-3b)).
α-Hemiacetal (*erythro*) (14%): *δ* [ppm] = 7.45 (d, $^{3}J_{H-1,OH}$ = 5.5 Hz, 1H, OH); 7.41-7.21 (m, Ph); 5.94 ('t', $^{3}J_{H-1,OH}$ = 5.5 Hz, $^{3}J_{H-2,H-1}$ = 5.2 Hz, 1H, C*H*OH (H-1)); 3.82 (ddd, $^{3}J_{H-3a;H-2}$ = 12.7 Hz, $^{3}J_{H-3b,H-2}$ = 8.4 Hz, $^{3}J_{H-2,H-1}$ = 5.2 Hz, 1H, *CH*CH$_2$ (H-2)); 2.97 (dd, $^{2}J_{H-3a,H-3b}$ = 17.1 Hz, $^{3}J_{H-3a,H-2}$ = 12.7 Hz, 1H, C*H*H (H-3a)); 2.71 (dd, $^{2}J_{H-3a,H-3b}$ = 17.1 Hz, $^{3}J_{H-3b,H-2}$ = 8.4 Hz, 1H, CH*H* (H-3b)).

Aldehyde (25%): *δ* [ppm] = 12.3 (br, COOH); 9.65 (br, 1H, CHO (H-1)); 7.41-7.21 (m, Ph); 4.11 (br 't', $^{3}J_{H-3a,H-2}$ = 8.0 Hz, $^{3}J_{H-3b,H-2}$ = 5.8 Hz, 1H, *CH*CH$_2$ (H-2)); 3.04 (dd, $^{2}J_{H-3a,H-3b}$ = 16.8 Hz, $^{3}J_{H-3a,H-2}$ = 8.0 Hz, 1H, C*H*H (H-3a)); 2.62 (dd, $^{2}J_{H-3a,H-3b}$ = 16.8 Hz, $^{3}J_{H-3b,H-2}$ = 5.8 Hz, 1H, CH*H* (H-3b));
Enol (1%): *δ* [ppm] = 12.3 (br, COOH); 9.20 (br, 1H, OH); 7.41-7.21 (m, Ph); 7.04 (s, 1H, =CH (H-1)); 3.38 (s, 2H, CH$_2$ (H-3)).

^{13}C-NMR (125 MHz, DMSO-d_6, 39.5 ppm):
β-Hemiacetal: *δ* [ppm] = 174.6 (CH$_2$*C*OO (C-4)); 139.1 (*i*-Ph); 129.2 (*m*-Ph); 128.1 (*o*-Ph); 127.7 (*p*-Ph); 104.6 (CHOH (C-1)); 49.0 (*C*HCH$_2$ (C-2)); 36.5 (CH$_2$ (C-3)).
α-Hemiacetal: δ/ppm = 176.7 (CH$_2$*C*OO (C-4)); 137.1 (*i*-Ph); 129.1 (*o*-Ph); 128.5 (*m*-Ph); 127.3 (*p*-Ph); 99.9 (CHOH (C-1)); 45.7 (*C*HCH$_2$ (C-2)); 32.0 (CH$_2$ (C-3)).

Aldehyde: *δ* [ppm] = 200.7 (CHO (C-1)); 173.1 (COO (C-4)); 136.1 (*i*-Ph); 129.4 (*m*-Ph); 129.3 (*o*-Ph); 128.1 (*p*-Ph); 54.1 (*C*HCH$_2$ (C-2)); 34.6 (CH$_2$ (C-3)).
Enol: δ/ppm = 173.5 (COO (C-4)); 142.4 (=CH (C-1)); 139.7 (*i*-Ph); 109.7 (=*C*CH$_2$ (C-2)); 31.9 (CH$_2$ (C-3)); not determined (*o*-, *m*-, *p*-Ph).

2.10 GC and HPLC chromatograms

Typical gas chromatography profile of biocatalytic reactions

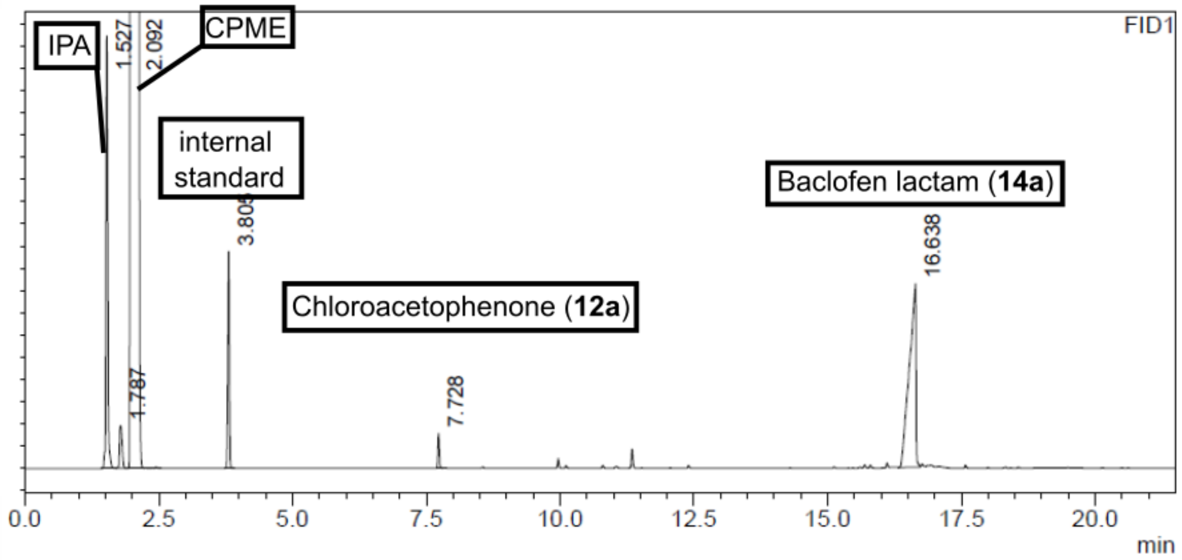

Fig. S7: Exemplary GC profile of an enzymatic reaction after basic extraction. (50 mM HEPES buffer, 250 mM IPA, 2.5 mM PLP, 30°C, 900 rpm, 48 h, 100 mM **2a**, 20 U/ml dwc, 1 ml, pH 10, extracted with CPME after NaOH treatment)

Chiral HPLC of hydrolyzed baclofen reaction products

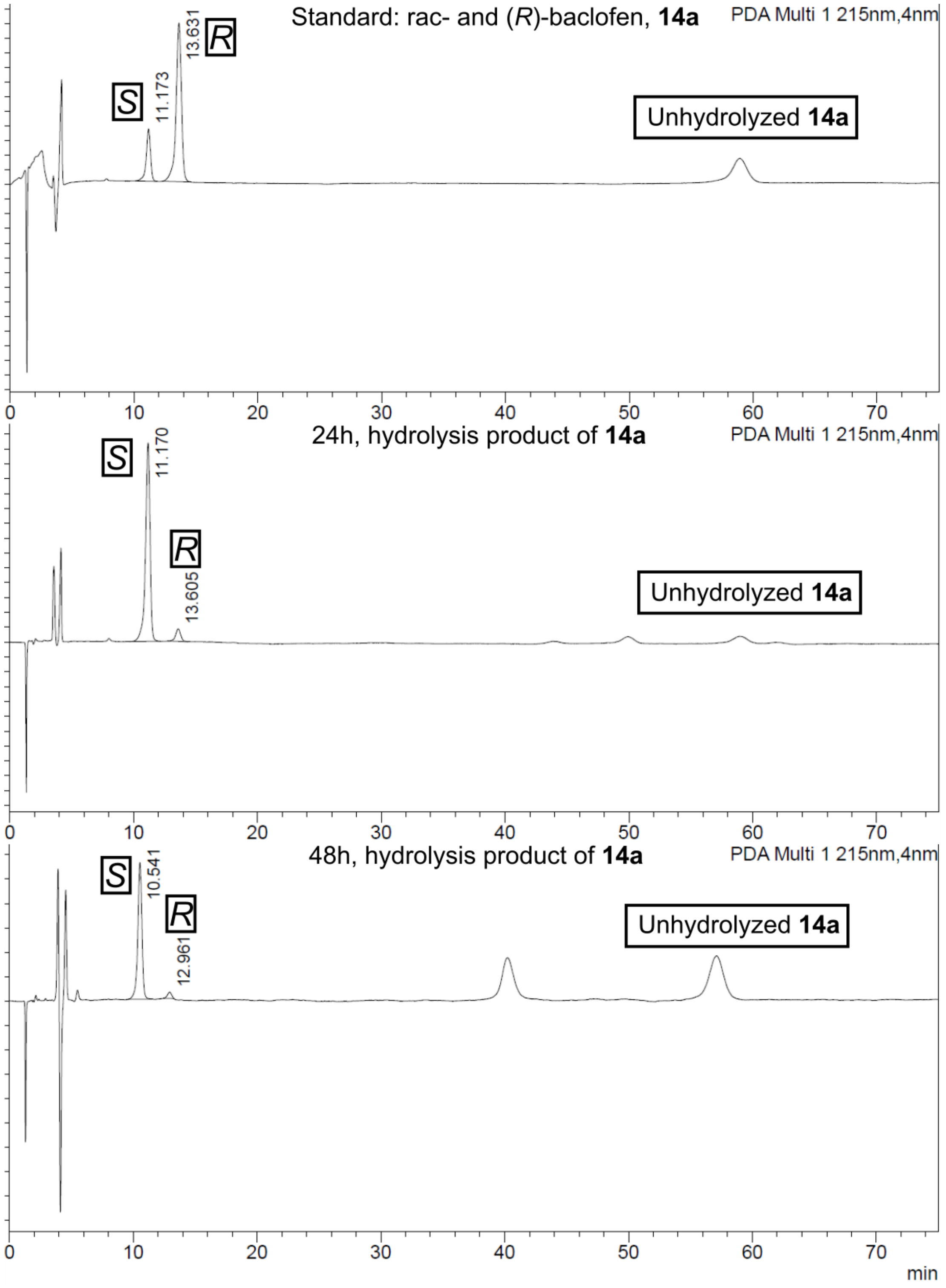

Fig. S8: Comparison of chiral HPLC spectra of the hydrolyzed baclofen lactam (**14a**). I. Standard of racemic baclofen with the addition of enantiopure (*R*)-baclofen and racemic **14a** (~60 min). II. Hydrolyzed **14a** isolated from 24 h fed-batch reaction. III. Hydrolyzed **14a** isolated from 48 h fed-batch reaction. (50 mM HEPES buffer, 250 mM IPA, 2.5 mM PLP, 30°C, 900 rpm, 24-48 h, 100 mM **2a**, 20 U/ml dwc, 1 ml, pH 10).

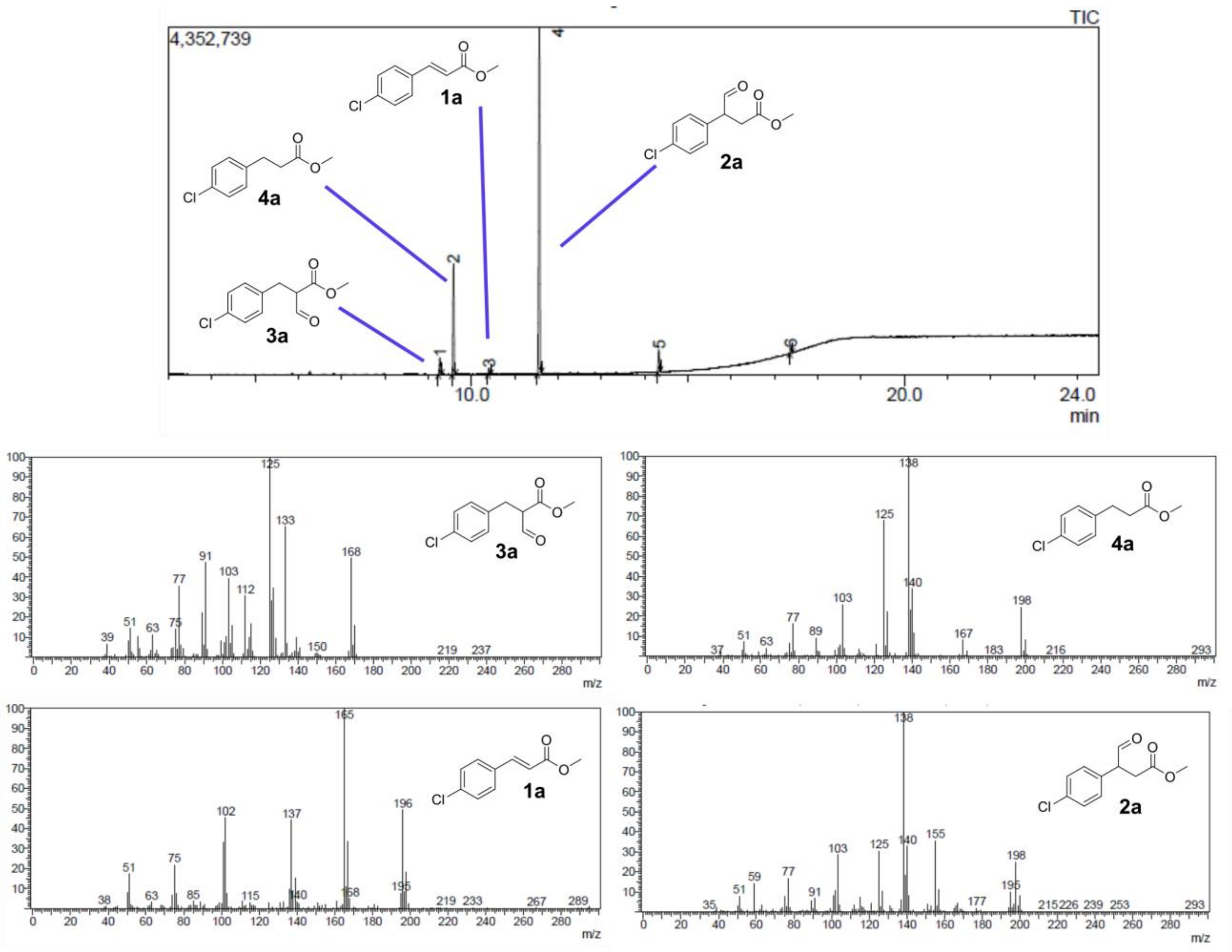

Fig. S9: Exemplary GC-MS characterization of a hydroformylation reaction for the synthesis of **2a**. By-product **4a**, substrate **1a** and the α-aldehyde **3a** are clearly distinguishable. The data was taken from Bork *et al.* [26] and completed for visualization.

2.11 IR spectra

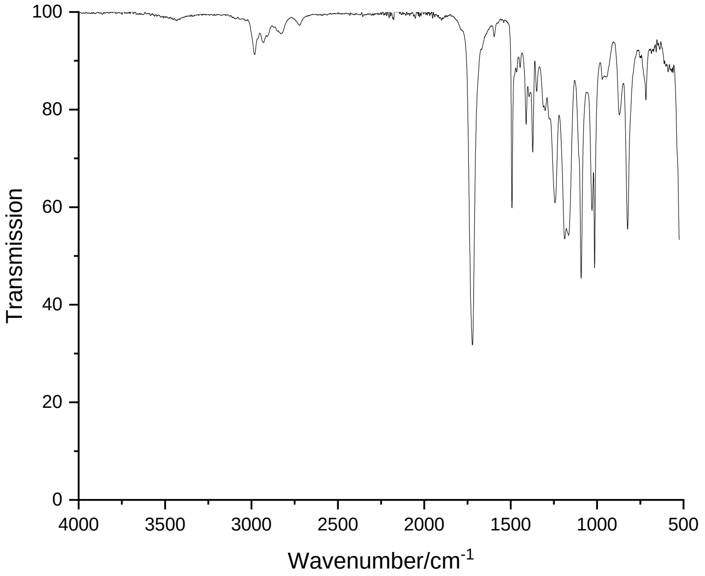

Fig. S10: IR spectra of ethyl 3-(4-chlorophenyl)-4-oxobutanoate (**2b**).

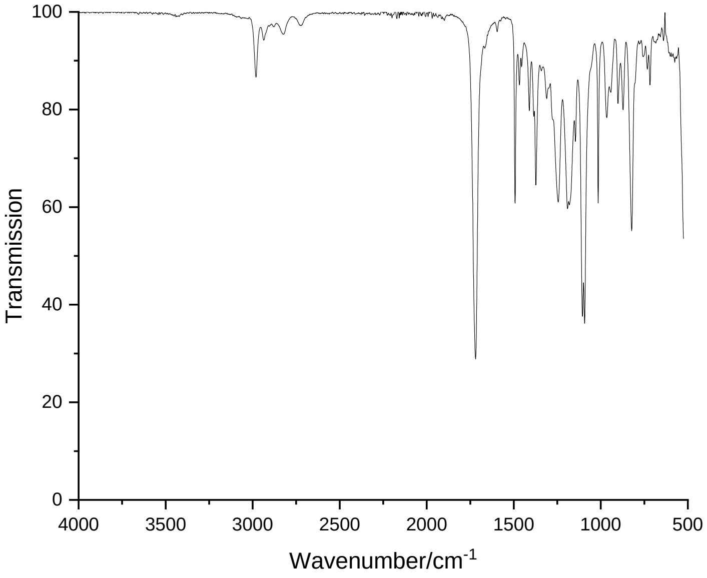

Fig. S11: IR spectra of isopropyl 3-(4-chlorophenyl)-4-oxobutanoate (**2c**)

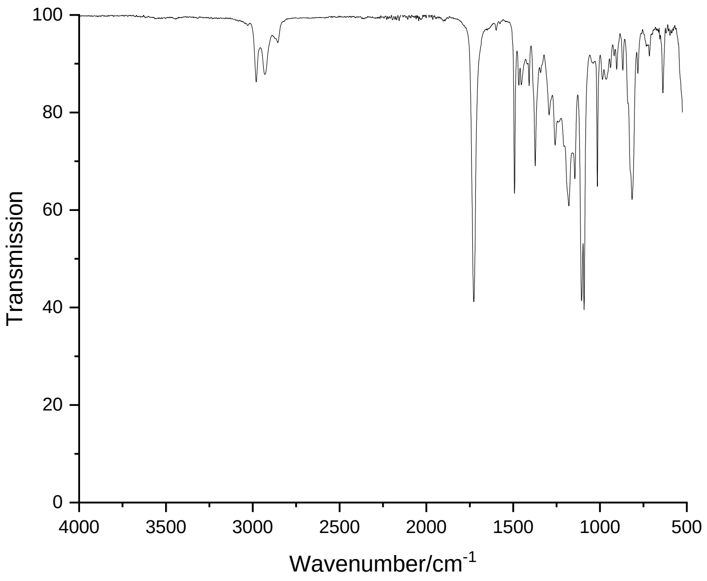

Fig. S12: IR spectra of isopropyl 3-(4-chlorophenyl)propanoate (**4c**).

2.12 Racemization study for the substrate 2a

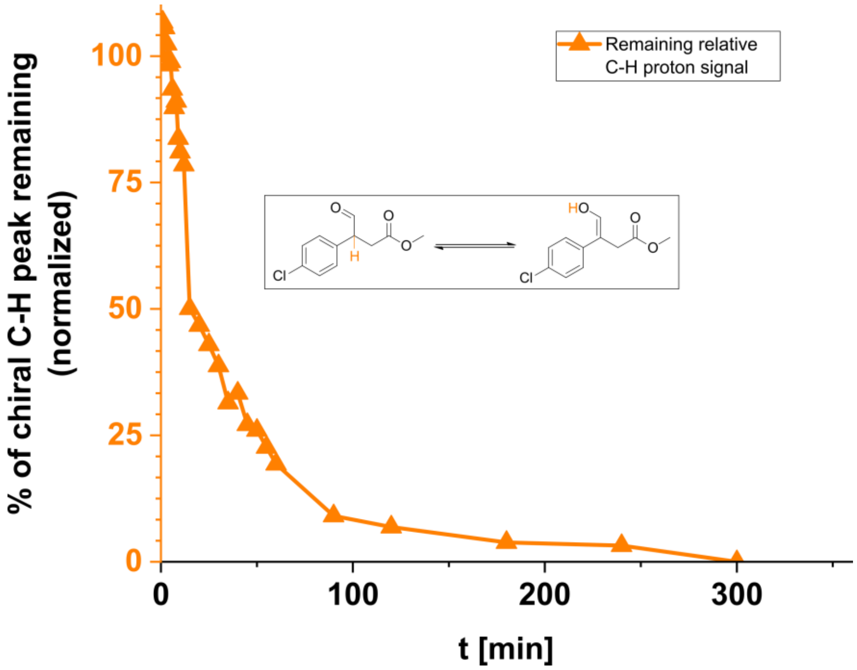

Fig. S13: Racemization experiment of **2a**. The integral of the C-H peak of the chiral centre proton was observed through NMR-scans at defined time points. Its relative area decrease through proton exchange due to keto-enol tautomerism was plotted over time. As can be seen, the racemization is fairly fast, since the proton exchange reaches its equilibrium after approximately 90 min. The NMR-spectra were recorded in a 50:50 mixture of D_2O:C_3D_6O.

<u>Experimental setup and result of the racemization study</u>

Racemic **2a** (22 mg) was dissolved in 1 ml of a 50:50 mixture of D_2O and deuterated acetone (C_3D_6O), which was used to increase solubility of **2a** in water for this experiment. Immediately after dissolving, the solution was transferred into an NMR tube and an NMR measurement was started. The NMR measurements were performed on a Bruker Avance 600 spectrometer.

At first, 100 ^{1}H-NMR scans were performed, at a rate of 1 scan every 5 seconds, capturing separate time moments of the proton exchange progression. After that, 300 scans were performed at a rate of 1 scan every 60 seconds (approximately 5 hours). Selected time points were picked out. The peak of the chiral C-**H** (see Figure S13) was integrated, normalized and plotted over time to yield its decrease over time. It was found, that after approximately 90 min the proton exchange (racemization) reached its equilibrium. The racemization process can thus be considered fairly fast and should not have a significant effect on the stereoselctivity of the transamination.

2.13 References

[1] R. H. N. Silva, A. C. M. Andrade, D. F. Nóbrega, R. D. de Castro, H. L. F. Pessôa, N. Rani, D. P. de Sousa, *Biomed Res. Int.* **2019**, 2019, 1–13.

[2] I. J. Montoya-Balbás, B. Valentín-Guevara, E. López-Mendoza, I. Linzaga-Elizalde, M. Ordoñez, P. Román-Bravo, *Molecules* **2015**, 20, 22028–22043.

[3] Y. Ogiwara, K. Takahashi, T. Kitazawa, N. Sakai, *J. Org. Chem.* **2015**, 80, 3101–3110.

[4] V. V. Komnatnyy, K. M. Taveras, N. S. Nandurkar, S. T. Le Quement, M. Givskov, T. E. Nielsen, Eur. *J. Org. Chem.* **2015**, 2015, 3524–3530.

[5] A. E. Bosnidou, K. Muñiz, *Angew. Chem. Int. Ed.* **2019**, 58, 7485–7489.

[6] D. R. Kumar, R. S. Panigrahy, D. Ravi Kishore, G. Satyanarayana, *ChemistrySelect* **2019**, 4, 12111–12116.

[7] C. Salomé, H. Kohn, *Tetrahedron* **2009**, 65, 456–460.

[8] H. Bork, T. Rösler, M. Leutzsch, N. Wessel, A. J. Vorholt, H. Gröger, *Eur. J. Org. Chem.* **2025**, DOI: 10.1002/ejoc.202401115.

[9] P. Klahn, H. Erhardt, A. Kotthaus, S. F. Kirsch, Angew. *Chemie Int. Ed.* **2014**, 53, 7913–7917.

[10] Y.-F. Ao, S. Pei, C. Xiang, M. J. Menke, L. Shen, C. Sun, M. Dörr, S. Born, M. Höhne, U. T. Bornscheuer, *Angew. Chem. Int. Ed.* **2023**, 62, e202301660.

[11] M. Kollipara, P. Matzel, M. Sowa, S. Brott, U. Bornscheuer, M. Höhne, *Appl. Microbiol. Biotechnol.* **2022**, 106, 5563–5574.

[12] M. Kollipara, P. Matzel, U. Bornscheuer, M. Höhne, *Chem. Ing. Techn.* **2022**, 94, 1836–1844.

[13] M. Voss, D. Das, M. Genz, A. Kumar, N. Kulkarni, J. Kustosz, P. Kumar, U. T. Bornscheuer, M. Höhne, *ACS Catal.* **2018**, 8, 11524–11533.

[14] O. Sviatenko, N. Ríos-Lombardía, F. Morís, J. González-Sabín, K. Venkata Manideep, S. Merdivan, S. Günther, P. Süss, M. Höhne, *ChemCatChem* **2019**, 11, 5794–5799.

[15] M. Genz, C. Vickers, T. Van den Bergh, H.-J. Joosten, M. Dörr, M. Höhne, U. T. Bornscheuer, *Int. J. Mol. Sci.* **2015**, 16, 26953–26963.

[16] A. Nobili, F. Steffen-Munsberg, H. Kohls, I. Trentin, C. Schulzke, M. Höhne, U. T. Bornscheuer, *ChemCatChem* **2015**, 7, 757–760.

[17] F. Steffen-Munsberg, C. Vickers, A. Thontowi, S. Schätzle, T. Meinhardt, M. Svedendahl Humble, H. Land, P. Berglund, U. T. Bornscheuer, M. Höhne, *ChemCatChem* **2013**, 5, 154–157.

[18] L. Skalden, C. Peters, J. Dickerhoff, A. Nobili, H.-J. Joosten, K. Weisz, M. Höhne, U. T. Bornscheuer, *ChemBioChem* **2015**, 16, 1041–1045.

[19] S. Schätzle, F. Steffen-Munsberg, A. Thontowi, M. Höhne, K. Robins, U. T. Bornscheuer, *Adv. Synth. Catal.* **2011**, 353, 2439–2445.

[20] H. Kohls, M. Anderson, J. Dickerhoff, K. Weisz, A. Córdova, P. Berglund, H. Brundiek, U. T. Bornscheuer, M. Höhne, *Adv. Synth. Catal.* **2015**, 357, 1808–1814.

[21] M. S. Weiß, I. V. Pavlidis, P. Spurr, S. P. Hanlon, B. Wirz, H. Iding, U. T. Bornscheuer, *ChemBioChem* **2017**, 18, 1022–1026.

[22] A. W. H. Dawood, M. S. Weiß, C. Schulz, I. V. Pavlidis, H. Iding, R. O. M. A. de Souza, U. T. Bornscheuer, *ChemCatChem* **2018**, 10, 3943–3949.

[23] M. S. Weiß, I. V. Pavlidis, P. Spurr, S. P. Hanlon, B. Wirz, H. Iding, U. T. Bornscheuer, *Org. Biomol. Chem.* **2016**, 14, 10249–10254.

[24] I. V. Pavlidis, M. S. Weiss, M. Genz, P. Spurr, S. P. Hanlon, B. Wirz, H. Iding, U. T. Bornscheuer, *Nat. Chem.* **2016**, 8, 1076–1082.

[25] S. Schätzle, M. Höhne, E. Redestad, K. Robins, U. T. Bornscheuer, *Anal. Chem.* **2009**, 81, 8244–8248.

[26] H. Bork, H. Gröger, *Eur. J. Org. Chem.* **2025**, e202401116.
